# Phase equilibria modelling of trace element evolution in arc magmas: implications for petrogenesis and copper porphyry indicators

**DOI:** 10.1007/s00410-026-02297-x

**Published:** 2026-02-16

**Authors:** Caroline R. Soderman, Owen M. Weller

**Affiliations:** Department of Earth Sciences, University of Cambridge, Cambridge, CB2 3EQ UK

**Keywords:** Arc magma, Copper porpyhry, Phase equilibria modelling, Trace element partitionin

## Abstract

Trace element ratios in arc magmas are widely used to infer petrogenetic conditions, particularly those associated with porphyry-forming versus barren systems. However, quantitatively linking whole-rock signatures to pressure, water content, or redox conditions remains challenging, as trace elements are sensitive to mineral assemblage and compositions, which both evolve during fractional crystallisation. Here, we integrate a recently updated thermodynamic model suite appropriate for arc systems with dynamic (composition-, temperature-dependent) mineral-melt partitioning to track trace element evolution. Benchmarking the model against published experiments, including apatite saturation, shows that the methodology successfully reproduces phase assemblages and compositions. We simulate fractional crystallisation of an average primitive arc magma across mid- to lower-crustal pressures (4–10 kbar), 2–4 wt% initial H$_2$O, and a range of redox conditions ($\sim$2 log units $\Delta$FMQ). We demonstrate how mineral-specific vectors evolve in trace element ratio (e.g. Sr/Y, Dy/Dy*) and rare-earth element shape coefficient ($\lambda$) space. Results highlight that amphibole and garnet may produce overlapping $\lambda$-space vectors under deep, hydrous conditions—contrary to orthogonal vectors inferred using static (composition-, temperature-independent) partitioning. Multiple petrogenetic paths can yield similar whole-rock trace element outcomes, particularly with poorly constrained primitive melt compositions. High Sr/Y, for example, commonly associated with porphyry systems, can form without particularly deep, hydrous, or oxidised conditions, when dynamic partitioning behaviour is considered. Overall, our modelling framework enables evaluation of arc magma petrogenesis and trace element evolution, with implications for porphyry indicators.

**Supplementary Information:**

The online version contains supplementary material available at 10.1007/s00410-026-02297-x.

## Introduction

Linking the trace element signatures of arc magmas to the petrogenetic conditions that formed them (e.g. pressure, water content, redox state), has been the focus of widespread research and subsequent debate. This interest is driven, in part, by the observation that arc magmas associated with porphyry copper (Cu) deposits often exhibit distinct geochemical signatures compared to barren arc magmas, including high Sr/Y, high La/Yb, elevated Al$_2$O$_3$/TiO$_2$, and more listric rare-earth element (REE) profiles (Richards 2011; Loucks 2014; Chiaradia 2015; Barber et al. 2021; Nathwani et al. 2022). These characteristics have been attributed to extensive amphibole ± garnet fractionation and suppressed plagioclase crystallisation because: heavy REEs (e.g. Yb) and Y are highly compatible in amphibole and garnet relative to silicate melts; amphibole has a concave-down REE profile (i.e. enrichment in middle REEs); and plagioclase crystallisation, the major host of Al$_2$O$_3$ in arc magmas, will deplete Sr from the melt. Such assemblage characteristics have been linked to hydrous, oxidised magmas at lower crustal pressures ($\sim$6–12 kbar) because of the impact of these conditions on the stability of these key phases (e.g. Macpherson et al. 2006; Richards and Kerrich 2007; Rodriguez et al. 2007; Richards 2011; Loucks 2014; Chiaradia 2015; Profeta et al. 2015; Farner and Lee 2017; Barber et al. 2021; Chen et al. 2023; Large et al. 2024). Because porphyry systems provide the majority of the world’s Cu, as well as other metals such as molybdenum and gold, these trace element signatures are increasingly used as potential exploration indicators (Sillitoe 2010).

However, interpreting such patterns, particularly their relation to crystallising assemblages, remains challenging. Different combinations of fractionating minerals (as well as source processes) can produce similar trace element trends, complicating the identification of specific petrogenetic processes (e.g. Defant and Drummond 1990; Stern and Kilian 1996; Richards and Kerrich 2007; Chiaradia et al. 2009, 2009b; Richards 2011; Chiaradia 2015; Barber et al. 2021; Tatnell et al. 2023).

For example, La/Yb primarily reflects the slope of a REE pattern, and is increased by garnet or amphibole crystallisation. High Sr/Y can also be generated by amphibole and/or garnet fractionation (e.g. Richards (2011)), but may also be linked to lower crustal assimilation (e.g. Chiaradia (2015)). Consequently, parameters such as Dy/Dy* (Dy$_\textrm{N}$/(La$_\textrm{N}^{4/13}$ $\times$ Yb$_\textrm{N}^{9/13}$), where $_\textrm{N}$ signifies chondrite-normalised values) have been designed to quantify the curvature of the REE pattern—effectively middle REE depletion or enrichment relative to light REEs and heavy REEs—to capture features not represented by simple slope metrics (Davidson et al. 2013). This parameter has been used to identify garnet versus amphibole (± clinopyroxene) fractionation in arc magmas when combined with Dy/Yb (Davidson et al. 2007), because of the distinct trends of garnet versus amphibole crystallisation in Dy/Dy*–Dy$_\textrm{N}$/Yb$_\textrm{N}$ space (e.g. Davidson et al. 2013; Zhou et al. 2020). However, several recent studies have highlighted the greater sensitivity of lambda ($\lambda$) coefficients for resolving fractionating mineral assemblages, compared to trace element ratios that may have non-unique solutions (Barber et al. 2021; Anenburg and Williams 2022; Tatnell et al. 2023; Leong et al. 2023). The $\lambda$ coefficients quantify the shape of chondrite-normalized REE patterns using orthogonal polynomial fits, capturing variations in their shape beyond traditional ratio-based approaches (O’Neill 2016; Anenburg 2020; Anenburg and Williams 2022). Lambda coefficients have recently been used to evaluate the role of amphibole versus garnet fractionation in producing the chemical traits of porphyry-associated arc magmas, suggesting that garnet fractionation is not necessary to produce the trace element signature of Cu-porphyry systems (Barber et al. 2021; Tatnell et al. 2023; Leong et al. 2023; Hao et al. 2024).

Quantitatively linking observed geochemical signals to petrogenetic conditions requires a prediction of the evolution of melt and mineral abundance and composition under different crystallisation scenarios. Phase equilibria experiments can provide valuable information to answer this question (e.g. Alonso-Perez et al. 2009; Blatter et al. 2013; Nandedkar et al. 2014; Ulmer et al. 2018; Marxer et al. 2022, 2023; Blatter et al. 2023). However, experimental approaches typically only cover a restricted area of parameter space (e.g. in terms of pressure, water content, and/or redox state). Alternatively, thermodynamic phase equilibria modelling offers a complementary approach that can systematically evaluate how variable petrogenetic conditions influence the mineral assemblage and, when combined with trace element partitioning models, predict resulting melt trace element chemistry (e.g. Yang et al. 2023; Nathwani et al. 2024; Soderman et al. 2025). However, despite its potential, such modelling has not been widely applied to arc magmas, in part due to the inability to model amphibole stability in the commonly used rhyoliteMELTS software (Gualda et al. 2012).

In this study, we apply a recently updated suite of thermodynamic models (Green et al. 2025) that are appropriate for modelling hydrous sub-alkaline melts and include thermodynamic descriptions of all of the major arc-relevant phases, including amphibole. We first benchmark these models against published experimental datasets for arc-relevant compositions, showing that these models reliably reproduce observed mineral assemblages and phase compositions. We then use the validated models to explore how pressure, water content, and redox conditions affect the mineral assemblage and phase chemistry during fractional crystallisation of arc magmas. By integrating these results with temperature- and composition-dependent trace element partitioning models, we quantify mineral-specific vectors in trace element ratio spaces (e.g. Sr/Y, La/Yb, Dy/Dy*–Dy$_\textrm{N}$/Yb$_\textrm{N}$) and in $\lambda$-coefficients. We examine how these vectors evolve during fractional crystallisation and assess how they combine to influence melt trajectories. Finally, we compare modelled melt compositions to global arc datasets to evaluate the resolving power and ambiguity of trace element tools for interpreting the petrogenetic histories of mineralised and barren arc systems.

## Methods

### Phase equilibria modelling of fractional crystallisation

Phase equilibria modelling is carried out using MAGEMin (MAGEMin_C version 1.7.6; Riel et al. 2022). We use the ds6.36 thermodynamic dataset (Holland and Powell 2011), with the set of composition-dependent equations of state (*x*-eos) presented in Green et al. 2025 (following Holland et al. 2018, using individual *x*-eos from White et al. 2014[mu_W14]; Green et al. 2016[amp_G16]; Holland et al. 2018[ol_H18]; Tomlinson and Holland, 2021[spl_T21]; Holland et al. 2022[fsp_H22]; Weller et al. 2024[g_W24, cpx_W24, opx_W24, ilm_W24]; Green et al. 2025[liq_G25w, fl_G25, bi_G25]). We use an eleven-component hydrous model system: Na$_2$O–CaO–K$_2$O–FeO–MgO–Al$_2$O$_3$–SiO$_2$–H$_2$O–TiO$_2$–Fe$_2$O$_3$–Cr$_2$O$_3$. For all model bulk compositions, reported whole-rock chemistries (Table S1) are converted to this model system by ignoring additional elements (e.g. minor MnO, P$_2$O$_5$; Table S2). However, given the potential importance of P$_2$O$_5$ for affecting REE partitioning via apatite, we introduce P$_2$O$_5$ as a separate ‘layer’ so that apatite saturation can be predicted (see schematic of approach in Fig. S1, and method outlined below). Fractional crystallisation is simulated via stepwise cooling in fixed temperature intervals. At each step, the crystallised solids are removed from the system, and the residual liquid is used as the input composition for the next calculation.

#### Starting compositions and model inputs

We model two starting bulk compositions previously used for experiments, to validate our thermodynamic modelling approach against experimental data under known conditions relevant to arc magma petrogenesis. The compositions are of two natural high-Mg basaltic dyke samples (RC158c, RC156; Table S1) from the Italian Adamello batholith. The former dyke is thought to represent a near primary mantle melt (Hürlimann et al. 2016), with 17 wt % MgO and Fo$_\textrm{91}$ olivine, and has been used for a series of fractional crystallisation experiments at 10 kbar (Ulmer et al. 2018). The latter, from the same dyke generation, is thought to be derived from RC158c by $\sim$15 % olivine fractionation (Ulmer et al. 2018) and has been used in a series of 7 kbar fractional crystallisation experiments (Nandedkar et al. 2014). The results of these experiments underpin previous models of trace element behaviour in arc magmas (e.g. Barber et al. 2021; Tatnell et al. 2023). The composition RC158c is synthesised as P$_2$O$_5$-free by Ulmer et al. (2018), whereas that of RC156 is synthesised including P$_2$O$_5$ by Nandedkar et al. (2014), so we only consider the effect of P$_2$O$_5$ (via an apatite saturation surface) for the latter model equivalent (see Table 1).

For a model equivalent of the experiments, we adopt redox and hydration states consistent with the experimental conditions (Table 1). The experiments were buffered, targeting oxygen fugacity (*f*O$_2$) conditions of the nickel-nickel oxide (NNO) buffer, but with subsequently calculated *f*O$_2$ conditions where olivine was present of $\Delta$NNO 1.0 to $-$1.5 in Ulmer et al. 2018 and 1.0 to $-$1.2 in Nandedkar et al. 2014. Therefore, we perform model calculations at $\Delta$NNO+1, using the model buffering capability introduced by Weller et al. (2024). Initial melt H$_2$O content is set at 3 wt% to match the experimental starting conditions. We only model the oxidised experiments from Ulmer et al. (2018), as these are most relevant to arc settings and have been used in subsequent trace element studies. Following the experimental protocols and conditions, fractional crystallisation calculations are performed from 1230 $^{\circ }$C at 10 kbar for RC158c, and from 1180 $^{\circ }$C at 7 kbar for bulk composition RC156 (to capture the liquidus), with all solids removed from the assemblage at 30 $^{\circ }$C intervals.

To generalise our results, we also use an average composition of primitive arc magmas in equilibrium with mantle olivine calculated from the primitive arc magma compilation of Tatnell et al. (2023). Following their method, the magmas in equilibrium with mantle olivine are identified as those with Mg# 0.70 to $-$0.75, Ni 150–500 ppm, Cr $\le$ 1200 ppm and MgO > 8 wt%. The average composition, calculated from 123 measurements and given in Tables S1–2, is used as the representative primitive arc magma in this study.

For modelling this general case, both *x*Fe$^{3+}$ (molar Fe$^{3+}$/Fe$_\textrm{T}$) and H$_2$O are explored as variables. We vary initial *x*Fe$^{3+}$ from 0.10 to 0.26 (equivalent to a $\Delta$FMQ range at the liquidus from $\sim -0.5$ to $\sim +1.5$ at 7 kbar), and H$_2$O from 2–4 wt% (from the typical lower limit to the average of global mafic arc magmas; Plank et al. 2013), to investigate the effects of redox and water on mineral assemblages and trace element evolution. Unlike the experimental comparison model calculations that feature oxygen fugacity (fO$_2$) buffered to $\Delta$NNO+1 as outlined above, the calculations for this general case are unbuffered, to better reflect the behaviour of fO$_2$ in nature (Frost 1991). We explore a range of mid- to lower-crustal pressures (4–10 kbar), with each calculation initiated from the modelled liquidus temperature at the pressure of interest. Since the modelling approach is not constrained by experimental feasibility, pure fractional crystallisation is simulated using 1 $^{\circ }$C temperature intervals.

A summary of the parameters involved in the various model calculations described above is given in Table 1.

**Table 1:** Summary of parameters used in model runs presented in this study. U18 = Ulmer et al. (2018), N14 = Nandedkar et al. (2014). *See Table S2 for bulk compositions. Prim. = primitive. Frac. = fractionation. Unbuff. = unbuffered

Aim	Composition*	Pressure (kbar)	H$_2$O (wt%)	P$_2$O$_5$?	Redox	T steps ($^{\circ }$C) of frac	Figs
Benchmark (U18)	R158c	10	3.0	No	$\Delta$NNO+1	30	1, 2
Benchmark (N14)	R156	7	3.0	Yes	$\Delta$NNO+1	30	3, 4
Generalised results	prim. arc magma	4–10	2.0, 3.0, 4.0	Yes	Unbuff	1	5+

### Trace element modelling

To link a crystallising mineral assemblage to its impact on trace element evolution in arc magmas, knowledge of the mineral-melt partitioning behaviour ($D^\mathrm {mineral/melt}$) is required. Our phase equilibria modelling approach provides the mineral and melt compositions at each pressure and temperature point, allowing direct integration with parameterisations of $D^\mathrm {mineral/melt}$ that are sensitive to temperature, and mineral and/or melt composition (i.e. ‘dynamic’). Such parameterisations, with sensitivity to one or more of those variables, have been published for REEs and Y in all the major phases relevant to arc systems. In the main modelling presented here, we use dynamic parameterisations of $D^\mathrm {mineral/melt}_\mathrm {REE+Y}$ in clinopyroxene (Bédard 2014), olivine (Bédard 2005), orthopyroxene (Bédard 2025), plagioclase feldspar (Bédard 2023), amphibole (Shimizu et al. 2017), garnet (Meltzer and Kessel 2020) and magnetite (Sievwright et al. 2020). For all these minerals except olivine, the dynamic $D^\mathrm {mineral/melt}_\mathrm {REE+Y}$ parameterisations are lattice strain models, with temperature as a variable in all, and a variety of melt and/or mineral compositional parameters (see Supplement for further details). The dynamic $D^\mathrm {olivine/melt}_\mathrm {REE+Y}$ parameterisation is a regression-based fit, using melt MgO as the variable. In addition, we apply static partition coefficients (i.e. composition-, pressure- and temperature-independent) for phases where applicable dynamic models are currently unavailable (Tables S3, S4): for biotite (Were and Keppler 2021), ilmenite (Shepherd et al. 2022), quartz (Nash and Crecraft 1985), rutile (Foley et al. 2000; Klemme et al. 2005) and aqueous fluid (Yang 2019). While dynamic fluid-melt partitioning models exist (e.g. Yang 2019), they require an input of chlorine molality (since both chlorine content of the fluid and major element composition of the melt have been shown to significantly impact $D^\mathrm {fluid/melt}_\textrm{REE}$; Borchert et al. 2010). Since chlorine is not included in the model system, such dynamic parameterisations cannot be integrated into the modelling approach used in this study, but could be used in future extensions of this approach. To test the robustness and sensitivity of our results, we also consider alternative REE partitioning parameterisations for selected key phases: the model of Beard et al. (2019) for clinopyroxene, Bonechi et al. (2023) for amphibole, and Sun and Liang (2013) for garnet. These alternative parameterisations are discussed in detail in the Supplement. Strontium partitioning is usually considered separately to the REEs, because of its divalent rather than trivalent cation. Dynamic parameterisations of $D^\mathrm {mineral/melt}_\textrm{Sr}$ are used for olivine, clinopyroxene, orthopyroxene and plagioclase (Bédard 2005, 2014, 2023, 2025), whereas static values are used for amphibole, garnet, magnetite, ilmenite, biotite, rutile, and aqueous fluid (Jenner et al. 1993; Klemme et al. 2005, 2006; Borchert, 2009; Were and Keppler, 2021; Bonechi et al. 2023; Tables S3–S4). Although an approximately temperature- and composition-invariant $D_\textrm{Sr}^\mathrm {amphibole/melt}$ is supported by experiments down to 800 $^{\circ }$C (Nandedkar et al. 2016), no such data are available for garnet and so we additionally consider an alternative published $D_\textrm{Sr}^\mathrm {garnet/melt}$ (Jenner et al. 1993) to test the sensitivity of our results. Further detail on the choice and form of the *D*s used is given in the Supplement and in Tables S3–S4.

#### Apatite saturation and trace element partitioning

Phosphorus is not included as a component in the thermodynamic model system of Green et al. (2025), and therefore apatite stability cannot explicitly be modelled during fractional crystallisation. However, apatite could play a crucial role in controlling the REE budget of arc magmas, given its strong compatibility for REEs (Watson and Green 1981), and is produced in the P$_2$O$_5$-bearing 7 kbar experiments of Nandedkar et al. (2014). Therefore, to predict apatite saturation, we incorporate an additional calculation within the fractional crystallisation modelling (Fig. S1). We use the empirical saturation surface of Harrison and Watson (1984), as recast for apatite saturation temperature by Piccoli and Candela (2002), which describes the concentration of P$_2$O$_5$ required for apatite crystallisation as a function of melt composition (SiO$_2$) and temperature. This solubility equation is generally considered suitable when the melt is metaluminous rather than peraluminous (e.g. Bea et al. 1992; Pichavant et al. 1992; Yakymchuk and Acosta-Vigil 2019), and therefore is appropriate for arc settings, where most reported whole-rock compositions are metaluminous (Blatter et al. 2013; Turner and Langmuir, 2015; see Fig. S2). The evolving phosphorus content of the melt is calculated dynamically during fractional crystallisation using the same approach as for REEs: composition-dependent $D_\textrm{P}^{\mathrm {mineral/melt}}$ values are applied to olivine, clinopyroxene, orthopyroxene, and plagioclase feldspar using published parameterisations (Bédard 2005, 2014, 2023, 2025), while static partition coefficients are used for other phases (Tables S3–S4). When the concentration of P$_2$O$_5$ in the melt exceeds the saturation level, the excess P$_2$O$_5$ is used to calculate a weight fraction of apatite produced and is removed (assuming an average stoichiometric concentration of 41 wt% P$_2$O$_5$ in apatite, following Yakymchuk, 2017 and the equivalent methodology outlined by Yakymchuk, 2023 for zircon saturation). Consequently, after the point of apatite saturation, the concentration of P$_2$O$_5$ in the melt follows the apatite saturation surface. Other oxides that partition into the apatite, mostly CaO but also minor SiO$_2$, MgO, FeO and Na$_2$O (using the composition of average igneous apatite; Piccoli and Candela, 2002), are also removed from the system prior to the next fractional crystallisation step.

The REE content of the crystallising apatite is calculated using the temperature- and melt composition-dependent partitioning model of Jirku et al. (2025). Strontium partitioning in apatite, by contrast, is less well constrained. Early experimental studies suggested that $D_\textrm{Sr}^\mathrm {apatite/melt}$ is relatively insensitive to both temperature (from 950–1120 $^{\circ }$C) and melt composition (from 50–68 wt% SiO$_2$; Watson and Green, 1981). However, the recent lattice strain model by Ji and Dygert (2024) indicates that partitioning of divalent ions in apatite may vary systematically with apatite composition. Because this latter model requires apatite compositional inputs not available from our phase equilibria calculations, it cannot be directly applied here. As an alternative, we adopt the empirical observation that the $D_\textrm{Sr}$/$D_\textrm{Y}$ ratio in apatite remains approximately constant across a range of melt compositions (Nathwani et al. 2020, following Prowatke and Klemme, 2006). We therefore use a fixed $D_\textrm{Sr}$/$D_\textrm{Y}$ ratio of 0.61, calculated from an andesitic melt composition by Prowatke and Klemme (2006), to calculate Sr partitioning dynamically from the modelled $D_\textrm{Y}^\mathrm {apatite/melt}$ derived from Jirku et al. (2025).

## Experimental benchmarks

**Fig. 1:**
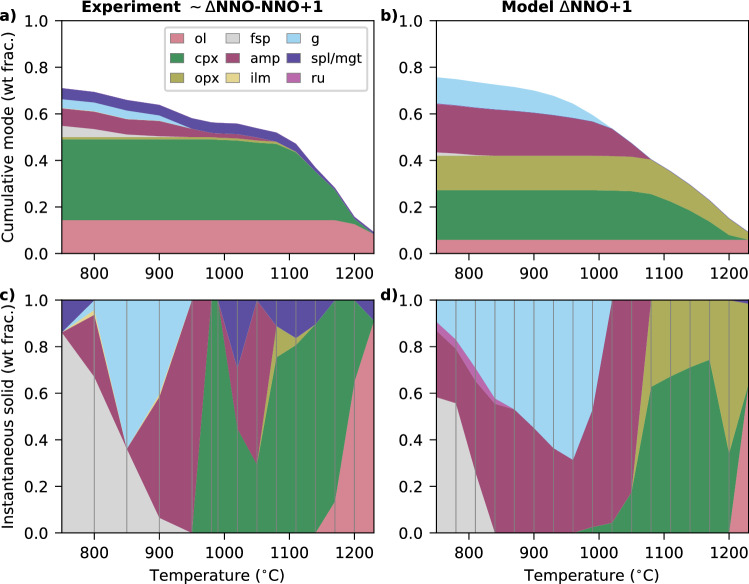
Comparison of the reported experimental assemblage from Ulmer et al. (2018) (**a**, **c**) to the model equivalent (**b**, **d**), with bulk composition RC158c (Tables S1–2). Model calculations and experiments are both at 10 kbar. Vertical lines in panels **c**, **d** demarcate the temperature of fractionation steps, with instantaneous modes interpolated between them. The modelled assemblage (panels **b**, **d**) is normalised to be anhydrous. Abbreviations are *amp* amphibole, *cpx* clinopyroxene, *fsp* feldspar, *g* garnet, *ilm* ilmenite, *ol* olivine, *opx* orthopyroxene, *ru* rutile, *spl/mgt* spinel/magnetite.

**Fig. 2:**
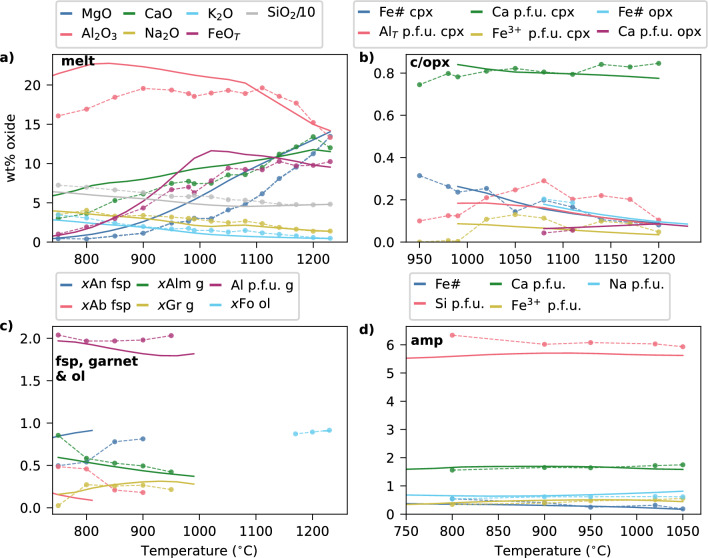
Comparison of the modelled phase compositions (solid lines) to reported experimental data (dots joined by dashed lines) from Ulmer et al. (2018), with bulk composition RC158c (Tables S1–2). Cations per formula unit (p.f.u.) are calculated using the AX activity-composition program (https://filedn.com/lU1GlyFhv3UuXg5E9dbnWFF/TJBHpages/ax.html). Model calculations and experiments are both at 10 kbar. Both experimental and model melt compositions are normalised to 100 wt% (anhydrous) with all Fe as FeO. Abbreviations as in Fig. 1, and additionally *Gr* grossular, *Alm* almandine, *Fo* forsterite, *An* anorthite, *Ab* albite.

**Fig. 3:**
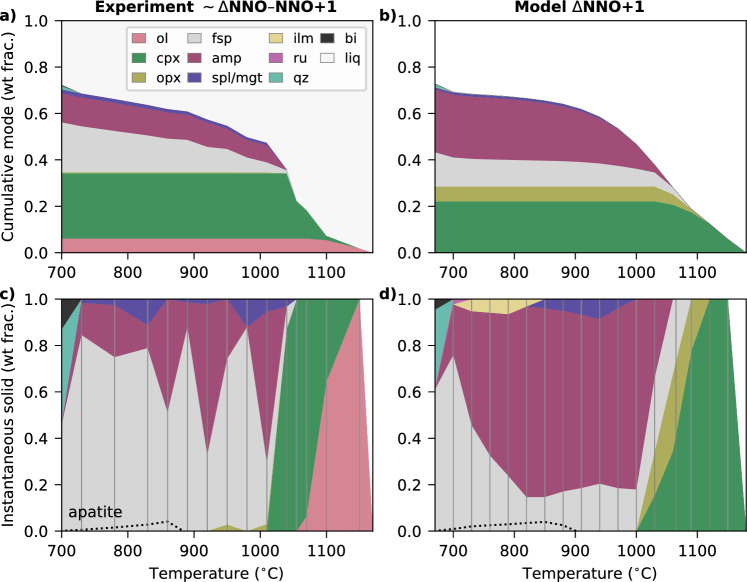
Comparison of the reported experimental mineral assemblage (**a**, **c**) to the modelled assemblage (**b**, **d**) from Nandedkar et al. (2014), with bulk composition RC156 (Tables S1–2). Model calculations and experiments are both at 7 kbar. Apatite is only shown on the instantaneous assemblage panels (**c**, **d**). It is shown as a dotted line because it is modelled separately outside the thermodynamic model system, i.e. the mode of the assemblage without apatite sums to 1, and the reported experimental assemblage (panel **c**) has therefore been renormalised in the same way to allow for a direct comparison. The modelled assemblage (panels **b**, **d**) is normalised to be anhydrous. Vertical lines in panels **c**, **d** demarcate the temperature of fractionation steps, with instantaneous modes interpolated between them. Abbreviations as in Fig. 1, and additionally *bi* biotite, *liq* liquid, *qz* quartz.

**Fig. 4:**
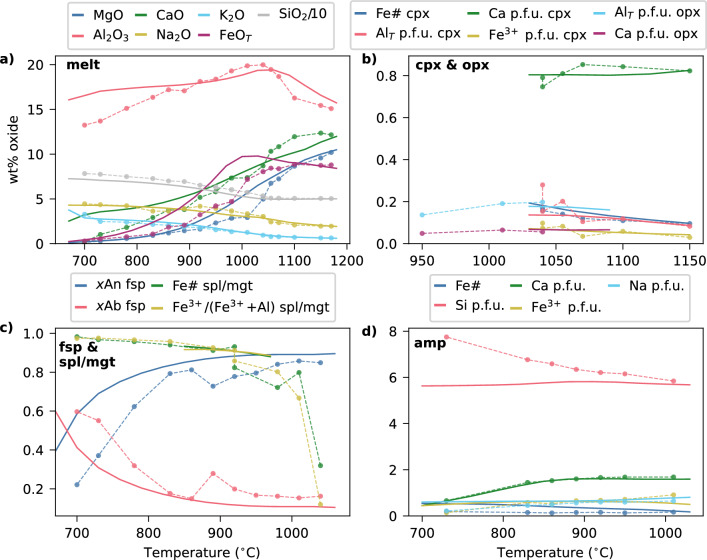
Comparison of the modelled phase compositions (solid lines) to reported experimental data (dots joined by dashed lines) from Nandedkar et al. (2014), with bulk composition RC156 (Tables S1–2). Model calculations and experiments are both at 7 kbar. Both experimental and model melt compositions are normalised to 100 wt% (anhydrous) with all Fe as FeO. Cations per formula unit calculated using the AX activity-composition program (https://filedn.com/lU1GlyFhv3UuXg5E9dbnWFF/TJBHpages/ax.html). Abbreviations as in Figs. 1, 2

For the published experimental data at 10 kbar (Ulmer et al. 2018) and 7 kbar (Nandedkar et al. 2014), we first describe the key experimental observations, and then compare our model results for the same conditions, considering both mineral assemblages (instantaneous and cumulative) and mineral compositions (Figs. 1, 2, 3, 4).

### 10 kbar fractional crystallisation

In the 10 kbar oxidised fractional crystallisation experiments of Ulmer et al. (2018), olivine and spinel are the liquidus phases at 1230 $^{\circ }$C (Fig. 1a, c). With decreasing temperature, clinopyroxene then minor orthopyroxene join the assemblage, olivine leaves the assemblage, and magnetite replaces spinel as the Fe-oxide phase. Amphibole appears and orthopyroxene is lost at 1050 $^{\circ }$C. Three near-replicate experiments were conducted at 980–990 $^{\circ }$C, and featured inconsistent phase assemblages and abundances. Ulmer et al. (2018) attributed this behaviour to variability in actual H$_2$O contents relative to target values, rather than disequilibrium. Two of these experiments (at 990 and 980 $^{\circ }$C) only crystallised clinopyroxene, in contrast to an amphibole-clinopyroxene-magnetite assemblage reported in a repeat at 990 $^{\circ }$C, and amphibole-bearing assemblages at higher (1020 $^{\circ }$C) and lower (950 $^{\circ }$C) temperatures. However, the amphibole-bearing replicate at 990 $^{\circ }$C had low inferred actual H$_2$O relative to the target value, whereas the other experiments had measured H$_2$O contents closer to the target. As a result, the clinopyroxene-only experiments were used to continue the fractionation series by Ulmer et al. (2018) (and are the results shown in Fig. 1a, c), leading to reduced cumulative amphibole and increased clinopyroxene in the interpreted fractionation sequence than would be inferred from experiments at immediately higher and lower temperature steps. With further cooling, garnet appears at 950 $^{\circ }$C, followed by the crystallisation of plagioclase and ilmenite. Amphibole remains in the assemblage at lower temperatures, except in one experiment at 850 $^{\circ }$C. Vapour bubbles were reported in all experiments below 1090 $^{\circ }$C despite the nominally added H$_2$O being lower than inferred solubility limits, and were attributed to CO$_2$ absorption. Apatite was not produced in these experiments because a P_2_O_5_-free system was used.

Compositionally, some of the key features of melt evolution identified by Ulmer et al. (2018) include an initial plateau in SiO$_2$, followed by a gradual increase below 1120 $^{\circ }$°C; a sharp inflection in Al$_2$O$_3$ at the point of plagioclase crystallisation (1140 $^{\circ }$C); and a slight initial rise in FeO$_T$, followed by a progressive decrease from $\sim$1140 $^{\circ }$C (Fig. 2a). Olivine is forsteritic (Fo$_{92}$ at the highest temperature), and the augitic clinopyroxene shows increasing Fe# during crystallisation but relatively uniform Ca content (Fig. 2b, c). Plagioclase feldspar evolves from anorthite-rich to approximately equal proportions of albite-anorthite, while garnet compositions show increasing almandine and grossular components until a sharp drop in grossular at the lowest temperature (Fig. 2c). Amphibole is initially pargasitic, with a slight increase in T-site Si at the lowest temperatures and overall constant total Na content (Fig. 2d).

In the model results, the first phases to stabilise are olivine, clinopyroxene, orthopyroxene and spinel, which appear simultaneously at the starting temperature of the experiments (Fig. 1b, d). Olivine and spinel are lost from the assemblage after the first crystallisation step, and orthopyroxene subsequently disappears when amphibole appears by 1050 $^{\circ }$C. With further cooling, garnet is stabilised by 990 $^{\circ }$C and clinopyroxene disappears. Finally, rutile and then feldspar appear. A free fluid phase is predicted to exsolve at 810 $^{\circ }$C (< 2 wt% of the instantaneous assemblage at any point), but is not shown in Fig. 1b, d because no equivalent experimental estimate of the amount of fluid in bubbles is provided. Compositionally, the modelled melt shows an initially flat SiO$_2$ profile followed by a gradual increase, an initial increase in Al$_2$O$_3$ which is suppressed after the onset of plagioclase crystallisation, and an increasing then decreasing FeO$_T$ (Fig. 2a). CaO and MgO decrease, while Na$_2$O and K$_2$O increase. The modelled orthopyroxene and augitic clinopyroxene show a notable increase in Fe# during crystallisation (Fig. 2b). Garnet initially increases, and then decreases, in its grossular component (Fig. 2c), while amphibole shows minimal compositional evolution (Fig. 2d).

Several key features of the 10 kbar experimental results are reproduced by the model. These include the rapid disappearance of olivine following orthopyroxene crystallisation, the subsequent loss of orthopyroxene upon amphibole appearance, and the onset of garnet crystallisation after $\sim$60 % total crystallisation (Ulmer et al. 2018). The modelled phase compositions and mineral stability temperatures are consistent (the former with the correct endmember of a given mineral, the latter within 50 $^{\circ }$C, the typical temperature error during modelling of major phases; Palin et al. 2016) with those observed for clinopyroxene, garnet and amphibole. The initial olivine composition is reproduced to within 1% of forsterite content. The composition of the initial anorthitic plagioclase is reproduced within 10 % (Fig. 2c), however, the modelled plagioclase is less stable than in the experiments, appearing 90 $^{\circ }$C later, and therefore undergoes less compositional evolution. Other notable discrepancies include the model producing rutile (up to 4 vol.% of the instantaneous solid assemblage) instead of ilmenite (up to 2 vol.% of the experimental instantaneous solid assemblage), less olivine and magnetite, and more orthopyroxene and amphibole, than observed experimentally (Fig. 1). These differences in assemblage lead to the evolved modelled melt at 750 $^{\circ }$C having higher Al$_2$O$_3$ and CaO contents than the experimental analogue (21.5 vs. 16.0 wt% and 6.0 vs. 2.6 wt%, respectively), as well as a higher maximum in melt FeO$_T$ during crystallisation (11.6 vs. 10.2 wt%). However, these discrepancies can be partly attributed to the variable experimental proportions of clinopyroxene and amphibole, as discussed above, and to the strong sensitivity of oxide abundances and compositions to redox conditions—which could not be independently confirmed by Ulmer et al. (2018) in experiments lacking olivine.

### 7 kbar fractional crystallisation

In the 7 kbar experiments of Nandedkar et al. (2014), the liquidus temperature is constrained to 1160 ± 5$^{\circ }$C, with olivine as the liquidus phase (Fig. 3a, c). Augitic clinopyroxene subsequently crystallises, and olivine is then lost by 1060 $^{\circ }$C. With further cooling, plagioclase feldspar and spinel crystallise simultaneously and clinopyroxene is lost, followed down-temperature by the appearance of amphibole, magnetite and minor orthopyroxene. Orthopyroxene disappears at 950 $^{\circ }$C, and apatite appears at 860 $^{\circ }$C. At the lowest temperature step (700 $^{\circ }$C), quartz and biotite also crystallise, accompanying plagioclase, magnetite and apatite, but no amphibole is present (Fig. 3a, c; Nandedkar et al. 2014). Variable phase modes are observed during cooling, particularly the absence of amphibole at 980 $^{\circ }$C and the associated high instantaneous plagioclase mode (Fig. 3c). The cause of this behaviour is not resolved by Nandedkar et al. (2014). However, they suggest that the relatively large temperature steps used in the experiments may lead to the disappearance and reappearance of phases along the fractionation path by jumping between stability fields (Nandedkar et al. 2014). In addition, many experiments exhibit CO$_2$-bearing vapour bubbles that additionally remove water from the melt, and measured H$_2$O contents in the run products are frequently lower than the amounts that were initially added to the capsules (Nandedkar et al. 2014), which could also be contributing to the observed variability in mineral modes between subsequent experiments.

Compositionally, the key features in melt evolution identified by Nandedkar et al. (2014) include an abrupt increase in SiO$_2$ at the onset of plagioclase and spinel crystallisation, accompanied by a switch to decreasing Al$_2$O$_3$ at the same point, and a general decrease in MgO and FeO$_T$ throughout crystallisation (Fig. 4a). Augitic clinopyroxene becomes increasingly Fe-rich with decreasing temperature (Fig. 4b), and shows a rise in Al content after the point of plagioclase crystallisation at 1050 $^{\circ }$C; crystals are sometimes sector-zoned. Orthopyroxene displays decreasing Ca and Al contents with progressive cooling (Fig. 4b). Plagioclase begins as highly anorthitic ($\sim$An$_\textrm{85}$) and becomes increasingly albitic with decreasing temperature (Fig. 4c). However, plagioclase compositions are notably variable, attributed by Nandedkar et al. (2014) to late-stage quench crystallisation and growth around refractory corundum grains used in the starting material, such that identifying equilibrium plagioclase compositions is not straightforward. Amphibole compositions evolve from pargasite at high temperatures to cummingtonite at the lowest temperatures, marked by a gradual increase in Si per formula unit (with a corresponding decrease in tetrahedral Al), and decreasing Ca and Na contents (Fig. 4d). The lowest temperature amphibole crystals also incorporate substantial Mn. Various Fe-Ti oxides are reported in these Cr-free experiments, from early hercynitic spinel, through to Ti-magnetite in more evolved experiments and one occasion of a coexisting ulvospinel with magnetite (Fig. 4c).

In our model equivalent of the experiment, the liquidus temperature of the arc magma is between the two modelled fractionation steps at 1150 and 1180 $^{\circ }$C, with clinopyroxene as the first phase to appear during fractional crystallisation, followed down-temperature by orthopyroxene, plagioclase feldspar, amphibole and magnetite, within a $\sim$200 $^{\circ }$C interval (Fig. 3b, d). Pyroxene disappears after amphibole appears. Apatite saturates at $\sim$900 $^{\circ }$C, followed by ilmenite replacing magnetite, with quartz and biotite forming when the system reaches $\sim$70 wt% total crystallisation. A free fluid phase is also predicted at 700 $^{\circ }$C (< 1 wt% of the instantaneous assemblage), but is not shown in Fig. 3b, d because there is no equivalent estimate of the amount of exsolved fluid from the experimental vapour bubbles (Nandedkar et al. 2014). The melt shows an initial increase in Al$_2$O$_3$ before a decrease at the point of plagioclase crystallisation, along with increasing SiO$_2$, Na$_2$O and K$_2$O and overall decreasing MgO, CaO and FeO$_T$ (the latter after the onset of magnetite crystallisation; Fig. 4a). Clinopyroxene and magnetite are predicted to remain compositionally similar across the 0–70 % crystallisation range modelled here (e.g. Mg-rich augitic clinopyroxene; Fig. 4b). The model predicts that the first amphibole and feldspar are initially calcic pargasite and anorthitic plagioclase respectively. As the melt becomes depleted in Ca during fractionation, later-stabilised amphibole becomes more Ca-poor and plagioclase becomes increasingly albitic (Fig. 4c, d).

By comparison, the modelled liquidus temperature and stability intervals for feldspar, amphibole, quartz and biotite are consistent with the experimental constraints (Nandedkar et al. 2014), within typical modelling errors of ±50 $^{\circ }$C for major phases (Palin et al. 2016). Additionally, modelled apatite saturation (and its modal abundance) is also consistent with the experiments, confirming the suitability of our approach to include apatite in arc magmas. Key changes in the modelled mineral assemblage during fractionation—such as the disappearance of clinopyroxene at the point of amphibole stabilisation, and the disappearance of amphibole once the melt reaches quartz and biotite saturation—are also consistent with the experimental observations (Fig. 3). However, our models do not predict olivine as the liquidus phase at 7 kbar; investigation at different pressures indicates that olivine is only stable in this bulk composition at $\lesssim$ 5 kbar. The model results also predict more orthopyroxene than observed in the experiments (6 vs. 0.5 cumulative wt %), and the temperature range over which the model predicts magnetite stability is significantly narrower than in the experiments, with ilmenite appearing to low temperature.

The same overall mineral assemblage produced in our model as in the experiments (with the exception of olivine and ilmenite) results in similar melt compositions—both in compositional trends and absolute values, which are within 10 % for most oxides (Fig. 4a). Discrepancies are most apparent in Al$_2$O$_3$ and CaO in the evolved melts, where the experiments show greater Al- and Ca-depletion than the model, and in FeO$_T$ at intermediate crystallisation stages, $\sim$850–1050 $^{\circ }$C. The first offset is explained by the model stabilising less Al- and Ca-rich plagioclase and more amphibole than observed in the experiments, largely due to the variable experimental modal abundances described above (which may in turn reflect varying H$_2$O in the experimental bulk compositions relative to the model), compared to the smoother modal progression produced by the model. Feldspar stability is also sensitive to water content (Holtz et al. 1995), which may contribute to the discrepancy given the uncertainties in actual experimental H$_2$O contents noted earlier. The second offset is explained by differences in Fe-oxide stability, which are highly sensitive to redox conditions (e.g. at $\Delta$NNO$-1$, ilmenite is stabilised before magnetite in the model) which cannot be verified in the experimental capsules in the absence of olivine (Nandedkar et al. 2014). The modelled mineral compositions replicate several key features of the experiments. These include augitic clinopyroxene with a slight increase in Fe$^{3+}$, Fe# and Al during modelled fractionation (Fig. 4b); initial plagioclase with $\sim$An$_{82}$ and a rapid increase in the albite component below $\sim$800 $^{\circ }$C (Fig. 4c); and high-temperature pargasitic amphibole with a sharp decrease in Ca between 730 and 830 $^{\circ }$C (Fig. 4d). There are some discrepancies between modelled and experimental plagioclase compositions throughout crystallisation, although these are difficult to evaluate given the uncertainties in identifying equilibrium compositions in the experiments. The modelled amphibole does not reproduce the experimental trend of increasing T-site Si (and corresponding decreasing Al) during crystallisation. This discrepancy may partly reflect the aforementioned differences in amphibole versus plagioclase modal abundance, and the resulting variation in melt Al$_2$O$_3$ toward the end of crystallisation. However, it may also indicate a limitation of the amphibole thermodynamic model at low temperatures, suggesting an area of investigation for future calibrations of this phase.

Alongside their experiments, Nandedkar et al. (2014) compared their results to modelling using rhyolite-MELTS (Gualda et al. 2012). They identified several discrepancies including a modelled liquidus temperature of 1260 $^{\circ }$C ($\sim$100 $^{\circ }$C higher than experimentally determined), the extensive modelled crystallisation of quartz ultimately leading to a silica-undersaturated system and the appearance of leucite, and a high modelled melt Al$_2$O$_3$ content ($\sim$24 wt%) at the end of crystallisation. The authors relate many of these issues to the known limitations of MELTS in modelling amphibole-bearing assemblages. In contrast, our model results show improvements in all of these areas.

### Summary of benchmarking

Overall, our model reproduces the key features of phase assemblages, mineral compositions, and melt evolution observed in both the 10 and 7 kbar experimental datasets (Figs. 1, 2, 3, 4). We suggest that some discrepancies, as discussed above, likely result from variable water contents and uncertain oxidation states in the experiments, leading to unconstrained differences with the model bulk composition. However, the over-stabilisation of orthopyroxene relative to olivine in both experimental comparisons may indicate a model limitation that should be assessed during calibrations of future thermodynamic models of these phases. Importantly, as discussed below, neither olivine nor orthopyroxene exerts a strong control on the REE systematics of arc systems, which are much more strongly controlled by amphibole, garnet, and clinopyroxene; all phases that exhibit better model matches to experimental constraints. Therefore, despite some discrepancies between reported experimental data and our modelled equivalents, we conclude that our phase equilibria modelling approach provides a suitable framework for simulating arc magma differentiation across a range of pressures. Consequently, we can use our approach to explore the broader effects of variables such as water content, pressure, and redox state on arc magma evolution.

## Average primitive arc magma: pressure, water and redox sensitivity

We now examine how predicted mineral assemblages and mineral and melt compositions vary as a function of pressure, water content, and redox conditions for an average primitive arc magma, and the resultant effects on trace element behaviour. These results allow us to assess the extent to which observed trace element signatures are diagnostic of specific petrogenetic conditions. The average primitive composition (calculated from the compilation of Tatnell et al. 2023) is slightly more Si- and alkali-rich, and Ca- and Mg-poor, than the experimental starting compositions described above (Table S1).

**Fig. 5:**
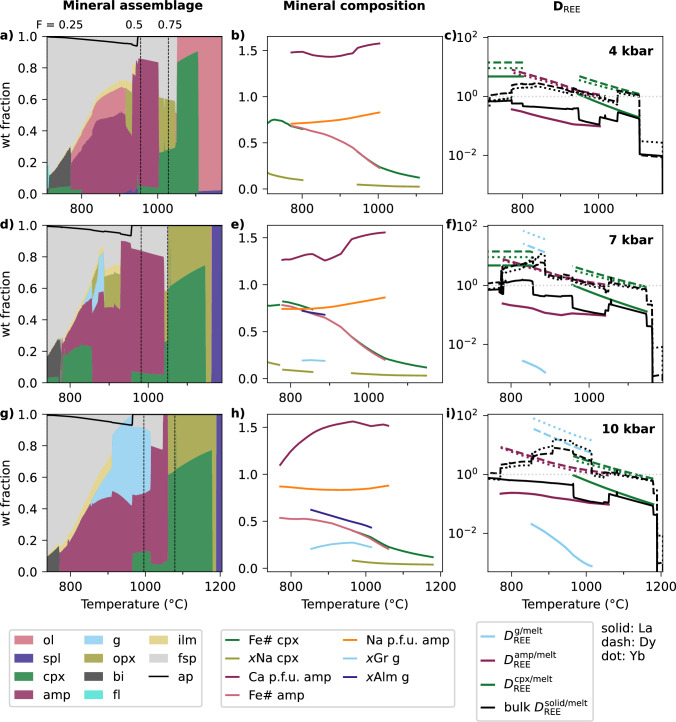
Instantaneous mineral assemblages (panels **a**, **d**, **g**), mineral compositions (panels **b**, **e**, **h**) and REE partitioning results (panels **c**, **f**, **i**) during fractional crystallisation of an average primitive arc magma (with 3 wt% H$_2$O and *x*Fe$^{3+}$ = 0.18) at 4 (a–c), 7 (d–f) and 10 (g–i) kbar. Compositions and *D* only shown for the minerals with key controls on trace element behaviour (amphibole, garnet, clinopyroxene). In panels **a**, **d** and **g**, vertical dashed lines show 75 and 50 wt% of the system remaining, and the x-axis is cut off at 25 % of the system remaining. As in Fig. 3, the coloured solid assemblage is shown normalised without apatite because apatite fraction is calculated outside of the thermodynamic model, and therefore apatite fraction is shown as a line on top of the rest of the assemblage. Abbreviations as in Figs. 1, 2, and additionally *ap* apatite, *fl* fluid.

First we explore the effect of varying pressure from 4 to 10 kbar, using an initial water content of 3 wt% H$_2$O and *x*Fe$^{3+}$ = 0.18 (Fig. 5). The pressure range covers typical mid- to lower-crustal depths relevant to arc magma fractionation (e.g. Chiaradia et al. 2025), and the redox conditions and initial water content were chosen to match those reported for the experimental composition RC156 from Nandedkar et al. (2014) described above. We then evaluate the influence of H$_2$O content from 2 to 4 wt% H$_2$O, and *x*Fe$^{3+}$ from 0.10 to 0.26 (Fig. 7, S1–2). The H$_2$O range spans the typical lower limit to the average of global mafic arc magmas (Plank et al. 2013), and the redox range was chosen to give a $\sim$2 log unit variation in $\Delta$FMQ at liquidus conditions. For example, at the 7 kbar liquidus, *x*Fe$^{3+}$ from 0.10 to 0.26 is equivalent to $\Delta$FMQ from $\sim -0.5$ to $\sim +1.5$, consistent with the range of $\Delta$FMQ in global arc magmas measured by XANES (mean of +0.96; Cottrell et al. 2021). However, unlike the externally buffered experimental comparisons, the oxidation state for the average primitive arc magma is internally buffered by the phase assemblage, better reflecting behaviour in nature (Frost 1991). As a result, $\Delta$FMQ evolves during crystallisation relative to the liquidus values. In all scenarios, fractional crystallisation is modelled until 25 wt% residual liquid remains, reflecting the degree of crystallisation required to generate andesitic to granitic compositions and consistent with the extent achieved in the benchmark experiments down to $\sim$700 $^{\circ }$C.

### Pressure

At 4 kbar spinel and olivine (Fo$_{88}$) are the liquidus phases, followed by Mg-rich augitic clinopyroxene (Fig. 5a, b). These minerals dominate the stable assemblage for the first $\sim$20 % of fractionation, during which both olivine and clinopyroxene evolve toward more Fe-rich compositions. Beyond this point, plagioclase feldspar and minor orthopyroxene stabilise, and olivine disappears from the assemblage. Calcic amphibole appears when $\sim$65 % of the initial model melt remains, within a temperature interval of rapid modelled crystallisation. Apatite saturates after $\sim$50 % fractionation. Over the next 25 % of fractionation, the assemblage is dominated by increasing proportions of increasingly albitic plagioclase and decreasing proportions of increasingly Fe-rich, Na-poor amphibole. Olivine, orthopyroxene, ilmenite, magnetite, biotite and clinopyroxene join and leave the stable assemblage at various points during this final stage of modelled crystallisation (to 75 % total crystallisation), with a fluid phase also stable at the final step. Late-stage clinopyroxene is Fe-rich (Fe# > 0.65) with a higher aegirine component than earlier-formed clinopyroxene. Under these conditions, the modelled melt shows a relatively rapid initial increase in alumina saturation index (ASI = molar Al$_2$O$_3$/(CaO + Na$_2$O + K$_2$O) as a function of melt SiO$_2$, driven by early olivine and clinopyroxene stability. This trajectory shallows after modelled feldspar saturation, and the melt approaches peraluminosity (ASI > 1) by $\sim$ 70 wt% SiO$_2$ (Fig. S2). Further melt composition parameters are given in Fig. S2 for reference.

**Fig. 6:**
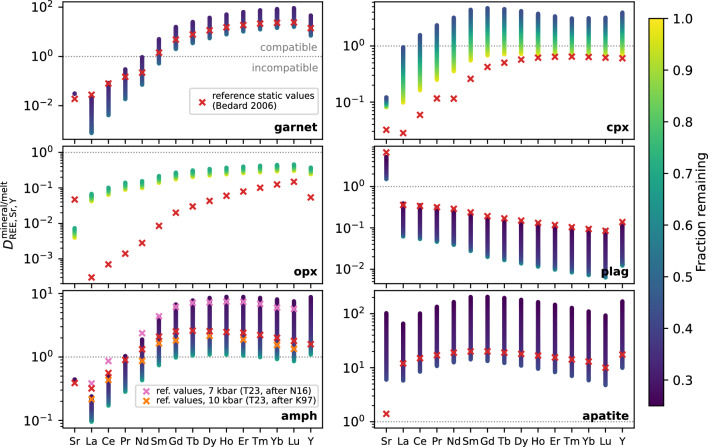
Comparison of modelled variability in $D_\textrm{REE,Sr,Y}^\mathrm {mineral/melt}$ during melt evolution at 10 kbar (3 wt% H$_2$O, *x*Fe$^{3+}$ = 0.18) to typically-used static values in crosses. The static examples shown are mostly taken from Bédard (2006) (red crosses; see Table S4), as used by Tatnell et al. (2023) to model the trace element evolution of arc magmas. Two additional $D^\mathrm {amphibole/melt}$ suites are shown because of different choices for modelled fractionation at 10 versus 7 kbar by Tatnell et al. (2023), following Klein et al. (1997) (orange crosses) and Nandedkar et al. (2016) (pink crosses); the values following Nandedkar et al. (2016) are actually the reported average during fractionation from a composition-dependent model.

The effect of the evolving assemblage on mineral/melt trace element partitioning (for clinopyroxene, garnet and amphibole) and on bulk trace element behaviour is shown for a representative light, medium and heavy REE (La, Dy and Yb, respectively) in Fig. 5c (higher pressures in panels f and i). An example of how the full suite of $D_\textrm{REE, Y, Sr}^\mathrm {mineral/melt}$ varies during a single liquid line of descent for a wider range of minerals is given in Fig. 6, and additionally highlights how our calculated values compare to typical static $D_\textrm{REE, Y, Sr}^\mathrm {mineral/melt}$ that are often used in other modelling studies. We show that $D_\textrm{REE}^\mathrm {mineral/melt}$ can vary by about an order of magnitude for a given element during fractional crystallisation, increasing as temperature decreases (Figs. 5, 6). For some minerals such as garnet or amphibole, reference static $D_\textrm{LREE}^\mathrm {mineral/melt}$ values from Bédard (2006) sit at the higher end of those predicted from the dynamic models, whereas $D_\textrm{HREE}^\mathrm {mineral/melt}$ sit at the lower end, therefore the use of dynamic partitioning behaviour will strongly affect the curvature of the REE patterns produced. This effect combines with the changing mineral assemblage to produce significant variability in bulk trace element behaviour during crystallisation. For example, at 4 kbar (Fig. 5c), for the first $\sim$ 50 % of fractionation La is strongly incompatible in the bulk solid assemblage ($D^\mathrm {solid/melt}$ $\lesssim$ 0.22), and Dy and Yb have maximum *D*s of $\approx$ 1. However, once amphibole becomes a dominant phase in the instantaneous assemblage, and as $D_\textrm{REE}^\mathrm {amphibole/melt}$ increases during crystallisation (due mainly to decreasing temperature, increasing melt SiO$_2$ and decreasing CaO, following the $D_\textrm{REE}^\mathrm {amphibole/melt}$ parameterisation of Shimizu et al. 2017), Dy and Yb become compatible in the bulk solid (*D* $\approx$ 1–3) while La remains incompatible. We note that $D_\textrm{REE}^\mathrm {amphibole/melt}$ does not depend on the Si content of the amphibole (Shimizu et al. 2017), and so while this parameter was identified as a potential discrepancy between experimental and modelled amphiboles in one of the experimental benchmarks (Fig. 4d), it will not significantly impact the resulting $D_\textrm{REE}^\mathrm {amphibole/melt}$.

As pressure increases, olivine is lost from the mineral assemblage (minor olivine at 7 kbar at 1161–1162 $^{\circ }$C, absent at 10 kbar; Fig. 5d, g) with a corresponding earlier stabilisation of both clino- and orthopyroxene. Plagioclase appearance is delayed by $\sim$15 % of fractionation from 4 to 10 kbar. Amphibole stabilises at higher temperatures (although at a relatively constant percentage of fractionation) with increasing pressure, and higher-pressure amphiboles evolve to lower Ca, lower Fe#, and higher Na than their lower pressure counterparts. As expected, garnet joins the stable assemblage at higher pressures, with minor garnet (< 1 cumulative wt %; locally up to $\sim$25 wt% of the instantaneous assemblage) at 7 kbar, increasing to 9 wt% at 10 kbar. Garnet becomes less almandine-rich and more grossular-rich with increasing pressure. The delay in feldspar saturation and increased clinopyroxene stability with increasing pressure results in modelled melt compositions reaching higher peak Al$_2$O$_3$ at 10 kbar than 4 kbar. Consequently, the modelled ASI rapidly reaches high values ($\sim$ 0.9) during fractionation at 10 kbar before its rate of increase (as a function of melt SiO$_2$) slows (Fig. S2). Overall, the mineralogical changes associated with increasing pressure in a hydrous system (suppressed plagioclase, stabilisation of amphibole, clinopyroxene and garnet) are consistent with experimental studies (e.g. Alonso-Perez et al. 2009; Marxer et al. 2022, 2023; Blatter et al. 2023). The resulting melt compositional trends, with particularly rapid increases in ASI at high pressure, are also consistent with experimental observations (e.g. Blatter et al. 2013; Nandedkar et al. 2014; Ulmer et al. 2018), although such isobaric trends are generally observed to poorly match natural arc rocks (Marxer et al. 2023). The consequences of the modelled changes in mineral chemistry on REE partitioning of each individual mineral are relatively small (Fig. 5c, f, i). However, the more dominant impact results from the changing mineral assemblage (mostly garnet abundance), with 10 kbar fractionation resulting in bulk $D^\mathrm {solid/melt}$ for Dy and Yb of up to 8.0 and 15.5 respectively (Fig. 5i), while bulk $D_\textrm{La}^\mathrm {solid/melt}$ is similar to the lower pressure cases.

### Water content

**Fig. 7:**
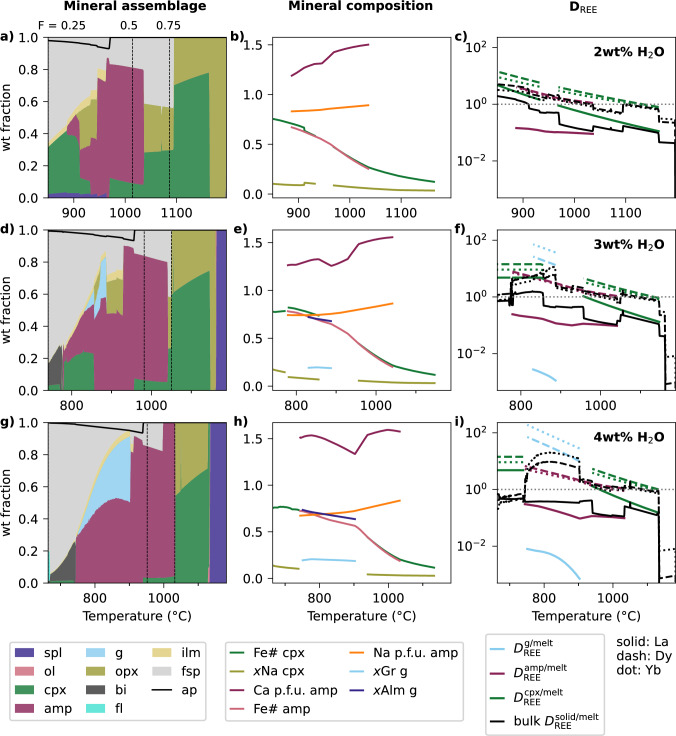
Instantaneous mineral assemblages (panels **a**, **d**, **g**), mineral compositions (panels **b**, **e**, **h**) and REE partitioning results (panels **c**, **f**, **i**) during fractional crystallisation of an average primitive arc magma at 7 kbar with 2 (a–c), 3 (d–f) and 4 (g–i) initial wt % H$_2$O. Panels **d**–**f** are identical to the same panels in Fig. 5, but are included again for easy comparison. Compositions and *D* only shown for the minerals with key controls on trace element behaviour (amphibole, garnet, clinopyroxene). In panels **a**, **d** and **g**, vertical dashed lines show 75 and 50 wt% fraction of the system remaining, and the x-axis is cut off at 25 % remaining. As in Fig. 3, the coloured solid assemblage is shown normalised without apatite because apatite fraction is calculated outside of the thermodynamic model, and therefore apatite fraction is shown as a line on top of the rest of the assemblage. Abbreviations as in Figs. 1, 5

When the initial H$_2$O content is varied from 2 to 4 wt% H$_2$O, at either 7 (Fig. 7) or 10 kbar (Fig. S3), several general trends in mineral assemblage are observed. The abundance of early spinel increases with higher H$_2$O, alongside both the increasing abundance and earlier appearance of amphibole. Garnet is increasingly stabilised with water content, for example garnet is absent with initially 2 wt% H$_2$O at 7 kbar, but present with increasing abundance at initially 3 and 4 wt% H$_2$O (Fig. 7a, d, g). This effect occurs because, in contrast, pyroxene and feldspar abundances decrease with increasing H$_2$O, liberating key components such as CaO required for garnet. Associated with the suppressed feldspar stability, melt Al$_2$O$_3$ reaches higher peak values as water contents increase (Fig. S2). Consequently, out of the petrogenetic conditions considered here, relatively dry and low pressure fractionation (4 kbar, 2 wt% initial H$_2$O) best matches the relatively shallow increase in ASI during fractionation observed in natural arc rocks (Fig. S2). While fully investigating the implications for the reported metaluminosity of intermediate arc rocks (e.g. Marxer et al. 2023) is beyond the scope of this paper, our results highlight how this modelling approach could be used to address such a question. Compositionally, at higher H$_2$O, the first garnet to stabilise is more grossular-rich than at lower H$_2$O, as it appears earlier from a less Ca-depleted melt. At 10 kbar with 4 wt% initial H$_2$O, where garnet is stable over a wide temperature interval, the final garnet is almandine-rich and grossular-poor, and the final amphibole exhibits a sharp drop in Ca content (Fig. S3)—similar to the behaviour observed in the experiments of Nandedkar et al. (2014).

The increased stability and wider compositional evolution of garnet and amphibole at higher water contents influences their $D_\textrm{REE}^\mathrm {mineral/melt}$ (Fig. 7b, e, h). Several melt and amphibole compositional terms, along with temperature, affect $D_\textrm{REE}^\mathrm {amphibole/melt}$ (Shimizu et al. 2017); in general *D* increases with melt SiO$_2$ and decreases with melt CaO. As Ca and Na contents in the M4 site of amphibole decrease, the ideal site size becomes smaller, better accommodating smaller REEs (e.g., Dy, Yb) than larger LREEs and causing $D_\textrm{La}^\mathrm {amphibole/melt}$ to plateau or decline. Consequently, during fractionation, $D_\textrm{Dy}^\mathrm {amphibole/melt}$ and $D_\textrm{Yb}^\mathrm {amphibole/melt}$ increase, whereas $D_\textrm{La}^\mathrm {amphibole/melt}$ increases more slowly and may even decrease where amphibole Ca content drops sharply in evolved melts at high pressures and high water contents (Fig. S3i).

In garnet, $D_\textrm{REE}^\mathrm {garnet/melt}$ increases with the ratio of Fe in garnet relative to the melt, as well as with decreasing temperature (Meltzer and Kessel 2020). As fractionation progresses and the garnet becomes more almandine-rich (Fig. 7e, h) while melt FeO decreases, $D_\textrm{REE}^\mathrm {garnet/melt}$ increases accordingly (Fig. 7f, i). In the high pressure, high H$_2$O case (10 kbar, 4 wt% initial H$_2$O), $D_\textrm{La}^\mathrm {garnet/melt}$ and $D_\textrm{La}^\mathrm {amphibole/melt}$ converge (Fig. S3i), implying that the crystallisation of these phases would result in similarly shaped REE profiles. On a bulk scale, the shift in mineral assemblage with increasing water content, particularly the increased garnet abundance, results in Dy and Yb behaving more compatibly in the bulk solid (Fig. 7c, f, i and S3). For example, at 10 kbar, the maximum bulk $D_\textrm{Dy}$ during crystallisation increases from 3 to 11.5 as initial H$_2$O increases from 2 to 4 wt% (Fig. S3).

### Redox state

With increasing *x*Fe$^{3+}$, olivine and garnet become less stable, whereas spinel and amphibole are stabilised (Fig. S4). For example, at 7 kbar with 3 wt% initial H$_2$O, the modelled cumulative weight fraction of garnet decreases from 0.7 cumulative wt% with *x*Fe$^{3+}$ = 0.1 to < 0.01 wt% with *x*Fe$^{3+}$ = 0.26, due to the concomitant decrease in Fe$^{2+}$ and increase in $X_{\textrm{Mg}}$ (Chinner 1960; Weller et al. 2013; Yakymchuk and Kirkland 2025). Additionally, the model predicts late-stage crystallisation of Fe-rich clinopyroxene, whose abundance decreases with increasing *x*Fe$^{3+}$. The behaviour of olivine, spinel and amphibole with increasing oxidation are consistent with experiments on arc magmas (Ulmer et al. 2018), although we note that since the oxygen fugacity in the experiments is externally buffered and these model calculations are not, results are not directly comparable.

The compositions of the major stable phases do not show any substantial variation with *x*Fe$^{3+}$, and therefore their $D_\textrm{REE}^\mathrm {mineral/melt}$ remain broadly unchanged (Fig. S4c, f, i). For the first $\sim$ 60% of fractionation, the overall mineral assemblages are also similar across redox conditions, resulting in no significant differences in bulk $D_\textrm{REE}^\mathrm {solid/melt}$. However, during the latter stages of modelled crystallisation, the changing mineral assemblage leads to resolvable variation in bulk $D_\textrm{REE}^\mathrm {solid/melt}$ at different redox conditions. At lower *x*Fe$^{3+}$ (0.10 and 0.18), garnet—which has $D_\textrm{La}$ < $D_\textrm{Dy}$ < $D_\textrm{Yb}$—can make up to 25 % of the instantaneous solid assemblage at 7 kbar (Fig. S4a). As a result, bulk $D_\textrm{Yb}^\mathrm {solid/melt}$ exceeds $D_\textrm{Dy}^\mathrm {solid/melt}$ (Fig. S4c). In contrast, at higher *x*Fe$^{3+}$, garnet remains a minor phase and does not significantly influence bulk *D* behaviour. Instead, amphibole dominates the assemblage, resulting in $D_\textrm{Yb}$ $\approx$ $D_\textrm{Dy}$ throughout modelled crystallisation (Fig. S4g).

### Sensitivity of trace element results to alternative *D* parameterisations

The three minerals that contribute most to variable trace element enrichment in arc magmas are garnet, clinopyroxene, and amphibole, owing to their modal abundance and ability to impart distinctive trace element signatures. Multiple $D_\textrm{REE}^\mathrm {mineral/melt}$ parameterisations are available for these minerals. We show and briefly discuss the trace element results using the alternative parameterisations in the supplement and Figs. S5–S6, and find that the overall behaviours are consistent with those presented above.

## Discussion

The trace element evolution of arc magmas is commonly linked to the crystallising mineral assemblage using geochemical proxies such as Sr/Y, Dy/Dy*, and $\lambda$ coefficients of the full REE profiles (e.g. Richards 2011; Davidson et al. 2013; O’Neill 2016; Tang et al. 2020; Barber et al. 2021; Tatnell et al. 2023). These parameters are widely employed to infer the fractionation of (or lack of) amphibole, garnet, and plagioclase during arc magma differentiation, and are central to models of the petrogenesis of these magmas, including the differences between Cu porphyry-bearing and barren systems. The pressure, water, and redox sensitivity of mineral stability and REE partitioning established above allows us to critically evaluate trace element vectors as mineralogical fingerprints, and explore the implications for identifying the petrogenetic conditions associated with arc magmas, including those capable of generating porphyry copper systems. All the modelling presented here assumes that equilibrium is achieved, both in the mineral assemblages produced, and their major and trace element composition. While in nature equilibrium may not always be reached, especially at low temperatures, our approach nevertheless provides a framework from which general interpretations can be made.

### Mineral variability in trace element vectors

**Fig. 8:**
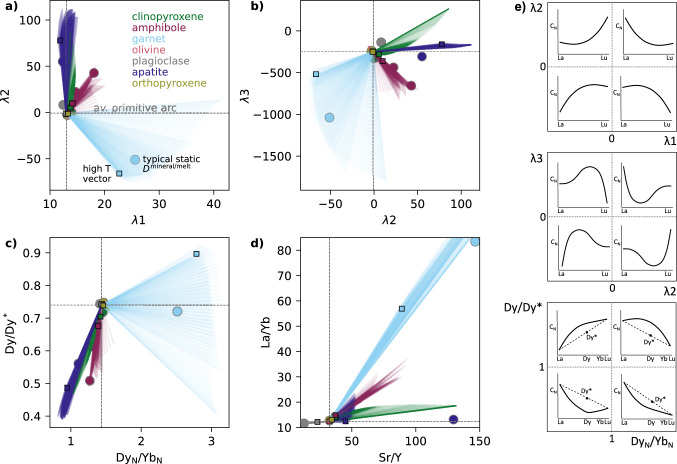
**a**–**d**) Modelled trace element vectors for the crystallisation of each phase from the primitive arc magma, across the range of considered petrogenetic conditions. Results are shown for 4 and 10 kbar, with 2, 3 and 4 wt% H$_2$O (with *x*Fe$^{3+}$ = 0.18), and *x*Fe$^{3+}$ = 0.10, 0.18 and 0.26 (with 3 wt% H$_2$O). The vectors show the effect of crystallising 20 wt% of each mineral (which each have varying $D_\textrm{REE}^\mathrm {mineral/melt}$ during fractionation depending on temperature and composition) for every 5 $^{\circ }$C of cooling in the modelled arc magma evolution. For reference, small squares show the highest temperature (i.e. initial) vector for the 10 kbar, 3 wt% H$_2$O, *x*Fe$^{3+}$ = 0.18 case for all phases, except olivine which is shown for the 4 kbar case. Circles show the result of using static $D^\mathrm {mineral/melt}$ from Bédard (2006) for all phases except amphibole (as used for modelling trace element evolution by Tatnell et al. 2023). Two amphibole circles are shown because Tatnell et al. (2023) used two different suites (Klein et al. 1997; Nandedkar et al. 2016) for two different pressures; the latter is a reported average from a compositional-dependent calculation, but no $D_\textrm{Sr}$ or $D_\textrm{Y}$ is given in either suite, so no amphibole circles are shown in panel d. The axes of panel d are truncated to allow smaller scale detail to be observed. (**e**) Fictive example REE patterns that schematically demonstrate what the parameter spaces in panels **a**–**c** represent (modified from Davidson et al. 2013; Barber et al. 2021, and $\lambda$-space patterns visualised using ALambdaR; Anenburg, 2020). Note that the average primitive arc magma composition used as the starting point in panels a–d is not the same point as the reference (0,0) or (1,1) lines in panel e, which divide up the parameter space into different sectors of REE profile shapes.

**Fig. 9:**
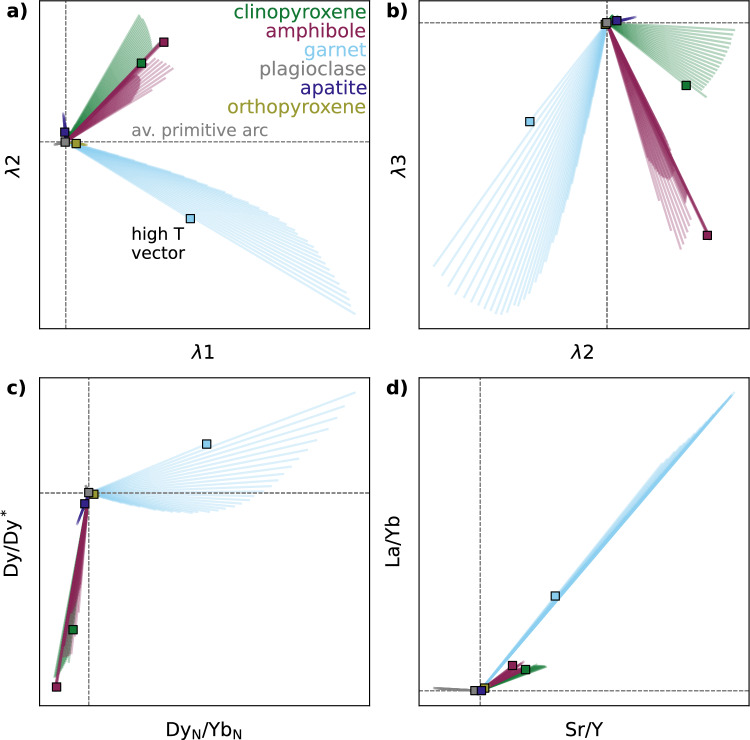
Scaled mineral vectors throughout a single liquid line of descent, at 7 kbar with 4 wt% initial H$_2$O and *x*Fe$^{3+}$ = 0.18. The direction range of vectors for each mineral reflects the composition- and temperature-dependent $D^\mathrm {mineral/melt}$ changing during modelled fractional crystallisation. Each vector has been scaled from the calculation shown in Fig. 8 by the contribution of the mineral at that point to the cumulative mineral assemblage (i.e. considering both the weight fraction of the mineral in the instantaneous solid assemblage, and the overall weight fraction of liquid left in the system relative to the start of the liquid line of descent). No quantitative scales are provided because only the relative vector lengths are relevant. The addition of all vectors at a given point in the crystallisation sequence throughout the liquid line of descent would yield the relevant paths for melt evolution in trace element space shown in Fig. 10

We use the modelled variation in $D_\textrm{REE, Sr, Y}^\mathrm {mineral/melt}$ during fractional crystallisation across a range of pressures, water contents and redox conditions (4 and 10 kbar with 2, 3 and 4 wt% H$_2$O at *x*Fe$^{3+}$ = 0.18, and *x*Fe$^{3+}$ values of 0.10, 0.18 and 0.26 at 3 wt% H$_2$O) to assess the predicted impact of individual mineral phases on commonly used trace element indicators. We first simulate the effect of crystallising 20 wt% of each mineral from the average primitive arc magma composition and calculate the resulting changes in trace element ratios and REE shape coefficients for all calculated phase compositions during the liquid lines of descent. Figure 8 presents the combined results for mineral compositions produced, in 5 $^{\circ }$C increments, across the full petrogenetic parameter space, with outlined squares highlighting the high-temperature vector for each mineral at 10 kbar, 3 wt% H$_2$O, and *x*Fe$^{3+}$ = 0.18 (except for olivine, which is shown at 4 kbar due to its absence at 10 kbar). Versions of Fig. 8a shown separately for each liquid line of descent are given in Fig. S7. Each mineral defines an array of ‘process vectors’ (O’Neill 2016), whose direction and magnitude vary with crystallisation conditions—reflecting how partitioning behaviour changes with temperature, melt composition, and mineral chemistry. Using the same crystallisation extent for each mineral allows for a direct comparison of the relative REE fractionation ‘power’ of different phases. However, in natural magmatic systems, the trace element evolution of a melt along a liquid line of descent is governed not only by the partitioning behaviour of each mineral (i.e. the direction and magnitude of the process vectors) but also by how much of each mineral actually crystallises. This interplay between partitioning behaviour and modal abundance is fundamental for interpreting whole-rock trends, which reflect the integrated effects of gradually evolving crystallising assemblages. To account for this effect, in Fig. 9 & S8 we show the vectors for a single liquid line of descent per figure, and scale each vector by the cumulative modal contribution of each mineral at each step of crystallisation. This approach provides a more realistic representation of the effective trace element influence of each mineral throughout fractionation, albeit constrained by just visualising one petrogenetic scenario per figure (unlike Fig. 8 that shows all scenarios).

#### $\lambda$ coefficients

Lambda ($\lambda$) shape coefficients (Fig. 8a, b) quantify the shape of chondrite-normalized REE patterns. $\lambda$1 represents the slope of the pattern (positive $\lambda$1 corresponds to a negative REE slope, i.e. LREE-enrichment), $\lambda$2 captures the quadratic curvature (positive $\lambda$2 is a concave-up pattern), and $\lambda$3 quantifies cubic curvature, or sinusoidality (with no direct physical significance, but allows for complex REE patterns to be represented; Anenburg and Williams, 2022; see Fig. 8). This approach to quantifying REE patterns has been used to identify garnet versus amphibole crystallisation in barren and porphyry Cu-bearing arc magmas (e.g. Barber et al. 2021; Leong et al. 2023; Tatnell et al. 2023; Gao et al. 2024). Garnet, for example, sequesters MREEs and HREEs, typically with $D_\textrm{MREE}^\mathrm {garnet/melt}$ < $D_\textrm{HREE}^\mathrm {garnet/melt}$, so its fractionation is expected to produce melt REE patterns that plot in the lower right quadrant of $\lambda$1–$\lambda$2 space and lower left of $\lambda$2–$\lambda$3 (O’Neill 2016). By contrast, amphibole, typically with $D_\textrm{MREE}^\mathrm {amphibole/melt}$ > $D_\textrm{HREE}^\mathrm {amphibole/melt}$ > $D_\textrm{LREE}^\mathrm {amphibole/melt}$ (Nandedkar et al. 2016; Shimizu et al. 2017), is predicted to generate process vectors approximately orthogonally to garnet, trending to the upper left quadrant of $\lambda$1–$\lambda$2 space and lower right of $\lambda$2–$\lambda$3. The end points of these expected vectors are shown by the light blue (garnet) and purple (amphibole) circles in Fig. 8a, b, calculated using the static *D* suites used for modelling in $\lambda$ space by Tatnell et al. (2023); see Table S4. Two amphibole points are shown because Tatnell et al. (2023) used different $D_\textrm{REE}^\mathrm {amphibole/melt}$ for 7 and 10 kbar fractionation (Klein et al. 1997; Nandedkar et al. 2016; Fig. 6).

In our model results, the high-temperature vectors for garnet and amphibole are approximately orthogonal in $\lambda$-space, with garnet showing a greater magnitude vector than amphibole for the same amount of crystallisation—consistent with the original predictions of O’Neill (2016). We also show vectors for the other modally important phases involved in arc magma differentiation (clino- and orthopyroxene, olivine, plagioclase) and for apatite (Fig. 8a, b). Although volumetrically minor, apatite can host substantial REEs due to high $D_\textrm{REE}^\mathrm {apatite/melt}$, especially in evolved, SiO$_2$-rich and CaO-poor magmas at low temperature (e.g. Watson and Green 1981; Jirku et al. 2025). The high-temperature apatite vector shows a large displacement in $\lambda$2, reflecting the concave-down REE pattern of apatite (Watson and Green 1981) and thus the concave-up melt pattern generated by its crystallisation, although the crystallisation of 20 wt % apatite used in these calculations is not realistic for natural arc systems. Clinopyroxene shows a similar high-temperature vector direction to amphibole but with smaller magnitude, while olivine and plagioclase have negligible vectors, again consistent with previous modelling (O’Neill 2016). The direction of these high-temperature vectors is relatively insensitive to the choice of $D^\mathrm {mineral/melt}$ parameterisation for amphibole, garnet and clinopyroxene (see Supplement), although the magnitude is variable (Fig. S6). The small vectors for olivine and orthopyroxene mean that, although in the experimental benchmarks we note a general overstabilisation of orthopyroxene relative to olivine in our models compared to experiments on arc magmas, there will be negligible impact on the resulting trace element evolution.

Although our results generally show the expected differences in vectors between minerals, Fig. 8 also highlights significant variability in vector direction and magnitude for some key minerals, driven by changes in REE partitioning behaviour both during a single liquid line of descent and across different petrogenetic conditions. For garnet, $\lambda$ vectors increase in magnitude down-temperature and shift from diagonal orientations at high-temperature to sub-horizontal (in $\lambda$1–$\lambda$2 space; Fig. 8a) or sub-vertical (in $\lambda$2–$\lambda$3 space; Fig. 8b) under certain conditions. These extreme vectors are associated with late-crystallising Ca-poor, Fe-rich garnet at 10 kbar with 4 wt% H$_2$O. In general, garnet vectors rotate to be less variable in $\lambda$2 as pressure and H$_2$O content increase, and as *x*Fe$^{3+}$ decreases (Fig. S7). This behaviour reflects the wider stability range of garnet under these conditions, which allows greater compositional evolution over a broader temperature interval (e.g. Fig. 5). Amphibole vectors show less directional variability than garnet, but still reflect changes in crystallisation conditions, particularly temperature (Fig. S7). For deep fractionation of a hydrous magma (10 kbar with 4 wt% H$_2$O), late-stage Ca-poor amphibole produces a vector that trends toward that of the simultaneously crystallising Ca-poor garnet (Fig. S7)—highlighting potential ambiguity in interpreting REE curvature signatures from single vectors alone. Such behaviour is not observed using an alternative $D_\textrm{REE}^\mathrm {amphibole/melt}$ parameterisation based on multiple regression analysis (Figs. S5, S6; Bonechi et al. 2023), but we consider the lattice strain model used in the main modelling here (Shimizu et al. 2017) to better capture the systematic effects of compositional variability because the same variables are used as inputs for each individual $D_\textrm{REE}^\mathrm {amphibole/melt}$ (see Supplement for discussion). As fractionation progresses, clinopyroxene vectors also increase in magnitude—eventually surpassing those of amphibole—and rotate slightly, to sub-vertical in $\lambda$1–$\lambda$2 (Fig. 8a) and to the upper right quadrant in $\lambda$2–$\lambda$3 space (Fig. 8b). The rotation reflects the transition from negatively sloping REE profiles of typical clinopyroxene in basaltic systems to more concave-down shapes characteristic of evolved rhyolitic melts (Bédard 2014).

#### Trace element ratios

Trace element ratios such as Sr/Y, La/Yb, and Dy$_\textrm{N}$/Yb$_\textrm{N}$, and the Dy/Dy* parameter, are also widely used to infer the involvement of specific minerals in the fractionating assemblage, and remain prevalent in arc magma studies despite several recent studies highlighting the greater sensitivity of $\lambda$ coefficients (e.g. Barber et al. 2021; Tatnell et al. 2023). Garnet and amphibole crystallisation are predicted to produce distinct trends in Dy/Dy*–Dy$_\textrm{N}$/Yb$_\textrm{N}$ space (lower left quadrant vectors for amphibole; upper right for garnet; Davidson et al. 2007, 2013). La/Yb primarily reflects the slope of the REE pattern, and is increased by both amphibole and garnet fractionation. Sr/Y in arc magmas is also affected by plagioclase, with the highest Sr/Y ratios in arc magmas—commonly associated with Cu porphyry systems (Loucks 2014)—generally interpreted to reflect fractionation under relatively deep, hydrous conditions, where plagioclase is suppressed and amphibole and/or garnet are stabilised (e.g. Richards and Kerrich 2007; Richards 2011; Richards et al. 2012; Chiaradia et al. 2009; Chiaradia 2015).

In Dy/Dy* versus Dy$_\textrm{N}$/Yb$_\textrm{N}$ space (Fig. 8c), our model results produce the expected behaviour: garnet fractionation increases Dy$_\textrm{N}$/Yb$_\textrm{N}$, while amphibole fractionation generally decreases it. The vector for high-temperature garnet crystallisation also increases Dy/Dy*, whereas amphibole decreases this metric. However, there is considerable variability in the vectors across the full pressure, temperature and compositional range modelled. For garnet, vectors rotate toward negative Dy/Dy* directions (relative to the initial composition) with decreasing temperature along a given liquid line of descent. This behaviour occurs because the partitioning parameterisation predicts that $D_\textrm{La}^\mathrm {garnet/melt}$ increases more than $D_\textrm{Yb}^\mathrm {garnet/melt}$ as temperature decreases. Because of this effect, garnet crystallising at higher pressure, higher H$_2$O and lower *x*Fe$^{3+}$ (i.e. conditions where the stability range of garnet is enlarged, hence garnet crystallises to lower temperatures) produces more steeply negative Dy/Dy* vectors than shallower, drier or more oxidised conditions. Amphibole vectors remain consistently negative in Dy/Dy* throughout crystallisation across all petrogenetic conditions considered. However, the Dy$_\textrm{N}$/Yb$_\textrm{N}$ vector for amphibole typically rotates from negative vector to either near-zero or even slightly positive. This change reflects convergence of $D_\textrm{Dy}^\mathrm {amphibole/melt}$ and $D_\textrm{Yb}^\mathrm {garnet/melt}$ at lower temperatures using the Shimizu et al. (2017) model, and is also observed using the Bonechi et al. (2023) model (Fig. S6). Apatite displays a large-magnitude vector in the lower left quadrant of Dy/Dy* versus Dy$_\textrm{N}$/Yb$_\textrm{N}$ space—comparable in length to garnet vectors for the same amount of fractionation (though this amount of apatite fractionation would be unrealistic). In contrast, olivine, plagioclase, and orthopyroxene generate negligible process vectors in this space.

Amphibole, garnet, clinopyroxene and orthopyroxene fractionation can all resolvably increase La/Yb (Fig. 8d). However, the magnitude of this effect is much larger for garnet than for the other minerals. Amphibole, clinopyroxene, orthopyroxene and apatite crystallisation also all increase Sr/Y, with garnet having the largest effect, and amphibole and clinopyroxene having a smaller effect but with similar magnitude to each other. As expected, plagioclase is the only mineral expected to decrease Sr/Y during its crystallisation. Overall in La/Yb versus Sr/Y space, there is relatively minor variability in the direction of the vectors for any given mineral across the conditions considered, with the exception of apatite. Using an alternative value of $D_\textrm{Sr}^\mathrm {garnet/melt}$ to the one used for Fig. 8 produces negligible difference compared to the much greater variability when using the REE parameterisations of Sun and Liang (2013) instead of Meltzer and Kessel (2020) (Fig. S6; supplementary discussion).

#### Dominance of individual minerals during fractionation

**Fig. 10:**
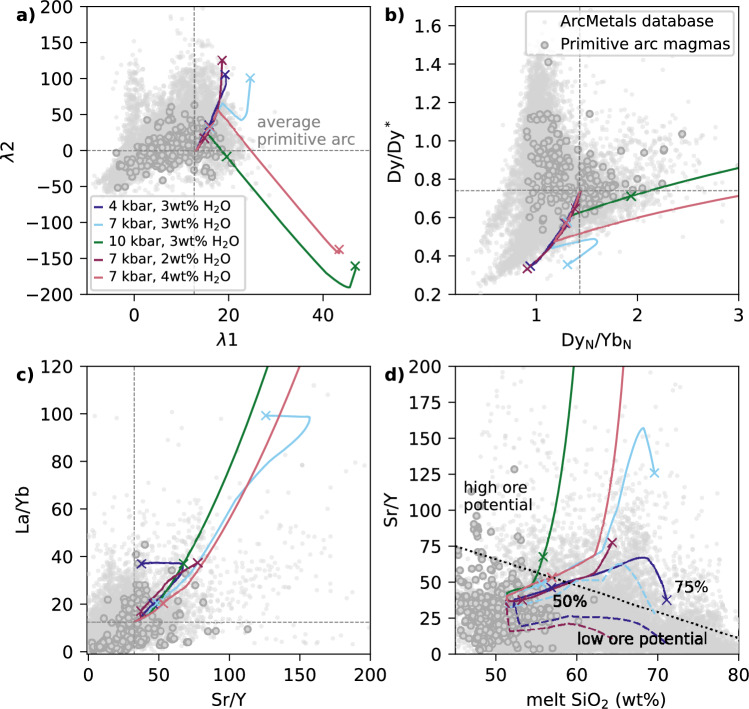
Modelled evolution of the trace element composition of a fractionating melt with varying pressure and water content. All calculations have *x*Fe$^{3+}$ = 0.18. Background dataset is the ArcMetals database (Barber et al. 2021), with outlined samples showing the primitive arc magmas compilation (MgO > 7 wt%, Mg# > 0.68) of Tatnell et al. (2023). Crosses mark 50 and 75 wt% fractional crystallisation. In (d), the dividing line between ‘high ore potential’ and ‘low ore potential’ magmas is from Loucks (2014). Dashed lines in panel d show selected results using static partition coefficient suites (see Fig. S10 for full results and Table S4 for partition coefficients).

In Fig. 9 we show the vectors for a single liquid line of descent at 7 kbar (with 4 wt% initial H$_2$O and *x*Fe$^{3+}$ = 0.18; i.e. Fig. 7g–i), scaled by the cumulative modal contribution of each mineral at each step of crystallisation. The relative magnitude of the vector for each mineral at a given temperature step therefore reflects its contribution to the overall vector for the bulk solid assemblage at that point in crystallisation, ultimately controlling the trace element evolution of the melt (e.g. Fig. 10).

We show that for these conditions, garnet fractionation exerts the strongest control on melt trace element composition across most of its crystallisation window, consistent with D$^\mathrm {garnet/melt}_\mathrm {M/HREE+Y}$ being at least an order of magnitude greater than other volumetrically-dominant phases (i.e. excluding apatite), and showing the strongest difference between partitioning behaviour of LREE and HREE (Fig. 6). In the example shown in Fig. 9, amphibole and clinopyroxene have approximately equal influence on the melt trajectory in $\lambda 2$–$\lambda 1$, Dy/Dy*–Dy$_\textrm{N}$/Yb$_\textrm{N}$, and La/Yb–Sr/Y space. However, amphibole dominates clinopyroxene in $\lambda 3$–$\lambda 2$. The relative magnitude of all these vectors will be affected partly by the petrogenetic conditions that influence mineral stability and composition. However, even in wetter magmas than considered here (e.g. < 7 wt% H$_2$O recorded in some mafic arc magmas; Plank et al. 2013; Rasmussen et al. 2022) crystallising at high pressure, garnet would still likely dominate the trace element budget over amphibole and clinopyroxene. This result is because of the D$^\mathrm {garnet/melt}_\mathrm {M/HREE+Y}$ characteristics described above, and because both garnet and amphibole can be stabilised by adding water (Fig. 7), not just amphibole. Quantifying such behaviour for fractional crystallisation paths relevant to particular arc settings is beyond the scope of this generalised study, but the modelling framework outlined here would be ideal for such investigations.

Within a single liquid line of descent, the greater sensitivity of $\lambda$ coefficients allows clearer discrimination between amphibole and clinopyroxene than using Dy/Dy*–Dy$_\textrm{N}$/Yb$_\textrm{N}$ ratios (Fig. 9). However, distinguishing these effects in whole-rock datasets—where trends reflect evolving instantaneous assemblages—is likely to be challenging. Most other minerals contribute negligible trace element vectors in all spaces, even when present in large modal abundance. For example, plagioclase feldspar, despite dominating late-stage crystallisation, has minimal impact on most considered metrics. However, it exerts a substantial effect on Sr/Y, producing a scaled negative vector comparable in magnitude but opposite in direction to amphibole and clinopyroxene. Thus, in the absence of garnet (either due to petrogenetic conditions or because the magma has evolved beyond its stability field) a dominance of feldspar over amphibole and clinopyroxene will drive Sr/Y downward (see Fig. 10).

### Identifying petrogenetic conditions from arc rock trace elements

The effects described above, specifically how variations in pressure, water and redox conditions influence the evolving mineral assemblage, mineral compositions, and the associated trace element process vectors, combine to allow forward modelling of melt evolution paths under differing petrogenetic conditions. Whole-rock datasets reflect snapshots of these paths, and are subsequently used to infer conditions for a given arc system (e.g. Barber et al. 2021; Tatnell et al. 2023). In Fig. 10, we illustrate these effects for the evolution of the average primitive arc magma composition along liquid lines of descent at 4, 7, and 10 kbar with 3 wt% initial H$_2$O, and at 7 kbar with 2 and 4 wt% initial H$_2$O (all at *x*Fe$^{3+}$ = 0.18). Results for variable redox conditions at 7 kbar are presented in Fig. S9. For each case, we track the melt evolution in (a) $\lambda$1–$\lambda$2, (b) Dy/Dy*–Dy$_\textrm{N}$/Yb$_\textrm{N}$, and (c) La/Yb–Sr/Y. In addition, we show the trajectory in (d) Sr/Y versus SiO$_2$ space, where an empirical threshold has been used to distinguish barren from Cu-porphyry-associated arc magmas globally (e.g. Loucks 2014; Barber et al. 2021). In all panels, we compare our modelled melt evolution paths to a global arc magma database (ArcMetals; Barber et al. 2021) and highlight the range of primitive arc magma compositions compiled by Tatnell et al. (2023). We note that the melt evolution paths shown here can broadly be translated around each panel for a different primitive starting composition (Tatnell et al. 2023), but that in detail changing the starting point of the paths in trace element space would also have an associated different in major element chemistry, therefore having small additional impacts on the crystallising mineral assemblage, compositions, and associated trace element vectors.

Our results highlight several key features of the predicted arc magma evolution. First, as expected from the strong influence of garnet—even when present at relatively low abundance (Fig. 9)—its crystallisation will produce detectable signatures in whole-rock trace element evolution. At the point of garnet crystallisation, the melt paths sharply deviate towards the lower right of $\lambda$2–$\lambda$1 (Fig. 10a), or towards the upper right in Dy/Dy*–Dy$_\textrm{N}$/Yb$_\textrm{N}$ (Fig. 10b). Such trends could, in principle, be used to identify garnet fractionation if the full crystallisation trajectory of a system were well preserved. However, apart from the most extreme cases (such as samples occupying the lower right of $\lambda 2$–$\lambda 1$ space or showing particularly high Dy$_\textrm{N}$/Yb$_\textrm{N}$), similar whole rock data in these spaces can also be produced by varying the extent of fractionation or the starting composition (i.e. moving the vectors around the range of trace element space covered by global primitive arc magmas).

Our results also highlight that a well-constrained primitive starting composition is crucial for confidently identifying the presence or absence of garnet during fractionation in any of the considered trace element spaces. Without this constraint, both garnet-bearing and garnet-free crystallisation pathways can be invoked to explain a given whole rock composition. This nuance explains why although modelled garnet-free or garnet-poor fractionation (e.g. at 4 or 7 kbar with 2–3 wt% initial H$_2$O) follows the lower envelope of Dy/Dy*–Dy$_\textrm{N}$/Yb$_\textrm{N}$ space, and effectively brackets the upper limit of most natural samples in La/Yb–Sr/Y space (supporting that extensive garnet fractionation is not generally required in arc systems; Barber et al. 2021; Tatnell et al. 2023), the results cannot unambiguously support or exclude garnet involvement from a global perspective. While our models reproduce the trace element composition of global arc magmas, robust interpretation of garnet fractionation would require arc-specific context, including (but not limited to) constraints on starting compositions and consideration of recharge and mixing processes (e.g. Tatnell et al. 2023). Such detailed arc-by-arc analysis, while beyond the scope of this study, has been addressed by others (e.g. Tatnell et al. 2023; Leong et al. 2023).

We also note that our models do not reproduce the high Dy/Dy*–low Dy$_\textrm{N}$/Yb$_\textrm{N}$ compositions observed in some magmas reported in the ArcMetals database (Fig. 10b), dominantly from the Pacific Izu-Bonin-Mariana and Tonga arcs. These high Dy/Dy*–low Dy$_\textrm{N}$/Yb$_\textrm{N}$ magmas are typically associated with elevated melt Cu contents, yet low porphyry mineralisation potential (Barber et al. 2021). Olivine and plagioclase fractionation can produce steeply positive vectors in Dy/Dy*–Dy$_\textrm{N}$/Yb$_\textrm{N}$ space (Fig. 9c; Davidson et al. 2013), but our models emphasise that these minerals can only exert a significant influence when amphibole, garnet, or clinopyroxene (which typically drive Dy/Dy* down) are crystallising in low abundances. Such behaviour (particularly suppressed amphibole and garnet, and elevated plagioclase crystallisation) could be generated by fractionation under shallow, upper crustal conditions—at lower pressure and water contents than explored here—but the very small magnitude vectors produced by these minerals (Figs. 8, 9) argue against this explanation being a viable mechanism to produce extremely high Dy/Dy* magmas. Alternatively, these compositions could derive from amphibole and clinopyroxene crystallisation of primitive melts with initially high Dy/Dy* and low Dy$_\textrm{N}$/Yb$_\textrm{N}$, and we note one such whole rock composition in the primitive arc magma database of Tatnell et al. (2023) (Fig. 10b). Resolving the origin of these signatures lies beyond the scope of this study but could be further explored with the modelling approach developed here.

The broader motivation behind interpreting trace element systematics in arc magmas has been to identify the petrogenetic conditions associated with arcs, with more recent interest in the origin of Cu porphyry-bearing versus barren systems. High Sr/Y and La/Yb have often been linked to early amphibole (± garnet) fractionation and plagioclase suppression in wet (and sometimes oxidised) magmas at depth, particularly in settings with thick crust (e.g. Macpherson et al. 2006; Richards and Kerrich 2007; Rodriguez et al. 2007; Richards 2011; Chiaradia 2015; Profeta et al. 2015). Such geochemical signatures are more commonly associated with mineralised than barren systems (Loucks 2014). Additionally, the observation that the mineralisation in porphyry systems typically occurs at the end of magmatic cycles when Sr/Y and La/Yb are highest is therefore interpreted to reflect a transition of the system to deeper crystallisation, potentially associated with crustal thickening, and/or higher magmatic water contents (Chiaradia et al. 2009; Nathwani et al. 2021; Chen et al. 2023; Large et al. 2024; Chiaradia et al. 2025). While the full range of processes influencing the formation of a Cu porphyry deposit will include more than just these parameters (including, for example, chlorine or sulfur contents and volatile exsolution, magma recharge, local stress regimes; e.g., Richards, 2003; Grondahl and Zajacz, 2022), the trace element signatures considered here are commonly used to understand broad petrogenetic systematics in Cu porphyry-bearing versus barren systems.

Our modelling confirms that both increasing pressure and increasing water content can produce higher Sr/Y and La/Yb in evolving arc magmas (Fig. 10c, d). However, we also find that significant increases in these ratios—high enough to reach the ‘high ore potential’ field of Loucks (2014)—can be achieved without extreme conditions, e.g. at mid-crustal pressures (4 kbar, $\sim$12 km) or with lower than average water contents (2 wt% H$_2$O in the primitive magma; Plank et al. 2013). Significant fractionation (> 50 %) under any of the considered conditions may instead be sufficient. This finding suggests that neither lower crustal fractionation nor particularly elevated water contents is a strict requirement for generating high Sr/Y and La/Yb signatures from a typical primitive arc magma, but does not rule out further elevation of these ratios from deeper or wetter conditions. As a comparison, in Fig. S10 we show how the results of Fig. 10 change when a suite of static $D_\textrm{REE, Sr, Y}^\mathrm {mineral/melt}$ values is used (primarily from Bédard, 2006, a commonly cited compilation; Table S4); selected results from Fig. S10d are also shown as dashed lines on Fig. 10d for ease of comparison. Substantial differences are observed relative to the dynamic model, particularly in Sr/Y, and to a lesser extent in other trace element ratios. Notably, the interpretation of trace element behaviour from using a static suite of $D^\mathrm {mineral/melt}$ is different from using the dynamic models: the use of static $D^\mathrm {mineral/melt}$ implies that elevated Sr/Y can only be achieved through deep, hydrous fractionation (e.g. $\ge$7 kbar, > 4 wt% initial H$_2$O)—an outcome not strictly required when using dynamic partitioning models. This difference arises for two main reasons.

First, the static $D_\textrm{Sr}^\mathrm {plagioclase/melt}$ lies at the upper end of the range produced by the composition- and temperature-dependent model of Bédard (2023)—nearly an order of magnitude higher than the values predicted for the initial plagioclase to crystallise in our models (e.g. Fig. 6). As a result, the bulk $D_\textrm{Sr}^\mathrm {solid/melt}$ exceeds 1 as soon as plagioclase joins the assemblage when using static values. In contrast, the dynamic model predicts a sharp rise in bulk $D_\textrm{Sr}^\mathrm {solid/melt}$ at the onset of plagioclase crystallisation, but it does not exceed one until later stages of fractionation. Second, the bulk $D_\textrm{Y}^\mathrm {solid/melt}$ is generally lower when using the static suite, because the $D^\mathrm {mineral/melt}$ for most minerals—particularly amphibole and clinopyroxene—fall at the lower end of the variability predicted by the dynamic models (Fig. 6). Therefore, the ratio of $D_\textrm{Sr}^\mathrm {solid/melt}$ to $D_\textrm{Y}^\mathrm {solid/melt}$ is typically higher along a given liquid line of descent with static values than with dynamic ones, leading to an over-prediction of Sr depletion relative to Y, and hence suppressed Sr/Y enrichment. These findings underscore the importance of incorporating temperature- and composition-dependent partitioning models when interpreting trace element behaviour, particularly for ratios such as Sr/Y that are highly sensitive to small changes in partitioning.

We also find that these trace element ratios, as well as Dy/Dy*–Dy$_\textrm{N}$/Yb$_\textrm{N}$ and $\lambda$ coefficients, show non-unique trajectories across pressure, water and redox conditions. For example, magmas evolving at 4 kbar with 3 wt% initial H$_2$O (dark blue line, Fig. 10) or 7 kbar with 2 wt% initial H$_2$O (purple line, Fig. 10) follow nearly identical paths through $\lambda$ and Dy/Dy*–Dy$_\textrm{N}$/Yb$_\textrm{N}$ space, and diverge only after $\sim$60–70 % crystallisation in La/Yb and Sr/Y. Similar overlaps occur between 10 kbar–3 wt% initial H$_2$O (green line, Fig. 10) and 7 kbar–4 wt% initial H$_2$O (pink line, Fig. 10) conditions. These similarities arise from the compensating effects of pressure and water on mineral stabilities, particularly those of plagioclase, amphibole, and garnet (Figs. 5, 6, 7). Consequently, interpreting high Sr/Y or La/Yb in arc magmas must account for the degeneracy between pressure and water content. For example, an increase in Sr/Y through time in an arc system could reflect deeper crystallisation with no change or even a decrease in the water content of primitive magmas, or no change in pressure but an increase in water content. Our results also show that redox conditions can influence these trace element trends: oxidation enhances amphibole stability but suppresses garnet (Fig. S4), leading to lower peak Sr/Y and La/Yb (Fig. S9d). However, the redox effect is secondary compared to the ranges observed for pressure or water content.

## Conclusions

We use phase equilibria modelling to explore how pressure, water content, and redox conditions affect mineral assemblages, phase compositions, and REE partitioning during fractional crystallisation of arc magmas. Comparison with reported experimental results confirms that the models reproduce key features of arc magma crystallisation and can therefore be used to both interpret and predict trace element evolution across a range of conditions.

Since the partitioning behaviour of trace elements in a given phase depends on temperature, mineral and/or melt composition, the trace element vectors associated with the fractionation of individual minerals are not fixed but vary both during fractionation and with variable petrogenetic conditions. For garnet and amphibole in particular, changing pressure, temperature and H$_2$O content influence their REE fractionation vectors due to evolving mineral compositions. For example, Fe-rich, Ca-poor garnet at high pressure and water content in an evolved arc magma produces a markedly different REE signature than grossular-rich garnet crystallising at higher temperatures. Similarly, amphibole vectors shift with composition and temperature, and could converge toward garnet-like behaviour in $\lambda$-space at the late stages of crystallisation under hydrous, high-pressure conditions. These potentially variable trace element vectors are not generally considered when interpreting the evolution of melt compositions.

Despite this complexity, our models successfully reproduce the majority of trace element signatures observed in natural arc magmas. We highlight that similar melt trace element compositions can emerge from different petrogenetic scenarios, with non-unique interpretations of the depth, initial water content or redox state of an arc system from the whole-rock chemistry. Parameters commonly associated with Cu porphyry-bearing arc systems (e.g. high Sr/Y and La/Yb) may not need to be generated under particularly deep, hydrous or oxidised conditions, as long as extensive fractionation (e.g. > 55 wt% at 4 kbar with *x*Fe$^{3+}$ = 0.18 and initial 3 wt% H$_2$O, or > 65 wt% at 7 kbar with *x*Fe$^{3+}$ = 0.18 and initial 2 wt% H$_2$O) can occur. This conclusion is only apparent when using dynamic trace element partitioning models. Overall, combined phase equilibria and trace element models such as these provide a powerful framework to test plausible petrogenetic conditions to disentangle the effects of pressure, water, redox, and mineralogy in arc magmas, with implications for understanding Cu porphyry fertility and broader magma evolution.

## Supplementary Information

Below is the link to the electronic supplementary material.

Supplementary file 1 (pdf 5381 KB)

Supplementary file 2 (xlsx 1206 KB)

Supplementary file 3 (xlsx 1117 KB)

Supplementary file 4 (xlsx 1134 KB)

## Data Availability

MAGEMin is free to download, along with example input files and scripts to run fractional crystallisation calculations such as those performed here (https://computationalthermodynamics.github.io/MAGEMin_C.jl/dev/).
