# Supplementary material for: Phase equilibria modelling of trace element evolution in arc magmas: implications for petrogenesis and copper porphyry indicators
Source: Contrib Mineral Petrol. 2026 Feb 16;181(3):17. doi: 10.1007/s00410-026-02297-x (PMC12909399; doi:10.1007/s00410-026-02297-x)
Supplement: Supplementary file 1 — (pdf 5381 KB) [file 410_2026_2297_MOESM1_ESM.pdf]

Supplementary Information

C. R. Soderman, O. M. Weller

Phase equilibria modelling of trace element evolution in arc magmas: implications for petrogenesis and copper porphyry indicators

Supplementary tables

Composition	SiO_2	TiO_2	Al_2O_3	Fe_2O_3	FeO	MgO	CaO	Na_2O	K_2O	P_2O_5	Cr_2O_3	MnO	H_2O
RC156$^{\alpha}$	48.43	0.68	15.19	1.64	6.65	10.13	11.57	1.86	0.59	0.10	–	0.15	3.00
RC158c$^{\beta}$	47.41	0.72	12.80	3.25	6.44	17.04	10.33	1.22	0.40	–	0.16	0.19	3.00
av. primitive arc$^{\gamma}$	50.90	1.05	14.99	–	8.34*	9.84	9.08	2.91	1.23	0.32	0.07	–	–

Table S1: Whole-rock data (wt%) for bulk compositions modelled in this study. Data sources: $^{\alpha}$Nandedkar et al. (2014), $^{\beta}$Ulmer et al. (2018), $^{\gamma}$average from Tatnell et al. (2023). *total Fe as FeO.

Composition	SiO_2	Al_2O_3	CaO	MgO	$FeO_{(T)}$	K_2O	Na_2O	TiO_2	O	Cr_2O_3	H_2O	buffer/$x Fe^{3+}$
RC156	46.68	8.63	11.95	14.56	6.55	0.36	1.74	0.49	2*	0.00	9.64	ΔNNO+1
RC158c	43.07	6.85	10.05	23.08	7.11	0.23	1.07	0.49	2*	0.06	9.09	ΔNNO+1
av. prim. arc	50.11	8.70	9.58	14.44	6.87	0.77	2.78	0.78	0.62	0.03	6.57 (2 wt%)	0.18
	48.52	8.42	9.27	13.98	6.65	0.75	2.69	0.75	0.60	0.03	9.54 (3 wt%)	0.18
	47.03	8.16	8.99	13.55	6.44	0.72	2.61	0.73	0.58	0.03	12.32 (4 wt%)	0.18
	48.39	8.40	9.25	13.95	6.63	0.75	2.68	0.75	0.33	0.03	9.51 (3 wt%)	0.10
	48.65	8.44	9.30	14.02	6.67	0.75	2.70	0.75	0.87	0.03	9.56 (3 wt%)	0.26

Table S2: Table S1 converted to thermodynamic model inputs (mol. %). $FeO_{(T)}$ = total Fe as FeO. For molar oxide inputs in MAGEMin, $x Fe^{3+} = 2^{*}O/FeO_{(T)}$. *For experimental comparisons that are buffered, excess O is provided to the system to allow for the formation of the buffer phase; see Weller et al. (2024) for further details.

Mineral	$D^{\mathrm{mineral/melt}}_{\mathrm{REE,Y}}$	$D^{\mathrm{mineral/melt}}_{\mathrm{Sr}}$	$D^{\mathrm{mineral/melt}}_{\mathrm{P}}$
Clinopyroxene	dynamic LSM (Bédard, 2014)	dynamic R (Bédard, 2014)	dynamic R (Bédard, 2014)
Orthopyroxene	dynamic LSM (Bédard, 2025)	dynamic R (Bédard, 2025)	dynamic R (Bédard, 2025)
Amphibole	dynamic LSM (Shimizu et al., 2017)	static; 0.44 (Bonechi et al., 2023)	static; 0.225 (Bédard, 2006)
Garnet	dynamic LSM (Meltzer and Kessel, 2020)	static; 0.031 (Jenner et al., 1993)	static; 0.184 (Bédard, 2006)
Plagioclase	dynamic LSM (Bédard, 2023)	dynamic R (Bédard, 2023)	dynamic R (Bédard, 2023)
Olivine	dynamic R (Bédard, 2005)	dynamic R (Bédard, 2005)	dynamic R (Bédard, 2005)
Magnetite/spinel	dynamic LSM (Sievwright et al., 2020)	static; 0.003 (Klemme et al., 2006)	static; 0.024 (Bédard, 2006)
Apatite	dynamic LSM (Jirku et al., 2025)	dynamic (tied to Y partitioning)	n/a
Ilmenite	static; Table S4 (Shepherd et al., 2022)	static; 0.004 (Klemme et al., 2006)	static; 0.002 (Bédard, 2006)
Fluid	static; Table S4 (Yang, 2019)	static; 0.24 (Borchert, 2009)	static; $1\times10^{-5*}$
Biotite	static; Table S4 (Were and Keppler, 2021, Y from Nash and Crecraft, 1985)	static (Were and Keppler, 2021)	static; 0.005 (Bédard, 2006)
Rutile	static; Table S4 (Foley et al., 2000, Klemme et al., 2005)$^{\alpha}$	static; 0.003 (Klemme et al., 2005)	static; 0.03 (Bédard, 2006)
Quartz	static; Table S4 (Nash and Crecraft, 1985)	static; $1\times10^{-5*}$	static; $1\times10^{-5*}$

Table S3: Details of the trace element partitioning behaviour used for the main modelling presented in the text. Further discussion of the models and values used is given in the supplementary text, and the static suites of D_{REE} are given in Table S4. LSM = lattice strain model. R = regression. *values of 1×10^{-5} are used where no other value is found. $^{\alpha}$values from experiment HD2_4D in Klemme et al. (2005) where available, and from Foley et al. (2000) otherwise.

	cpx^+	opx^+	g^+	amp^+	ap^+	pl^+	mgt^+	ilm^*/ilm^+	bi^*/bi^+	ol^+	fl^*	ru^*	qz^*
La	0.03	0.0003	0.03	0.32	12	0.36	0.015	0.002/0.02	0.61/0.02	$^{\alpha}1\times10^{-5}$	1.84	0.0001	0.016
Ce	0.06	0.007	0.08	0.56	15	0.34	0.016	0.003/0.01	0.32/0.03	3×10^{-5}	2.11	0.0001	0.014
Pr	0.12	0.001	0.15	0.90	17	0.32	0.018	0.0035^{β}/0.01	0.29/0.01	0.002	2.14^{β}	0.0001	$^{\alpha}1\times10^{-5}$
Nd	0.12	0.003	0.22	1.32	19	0.29	0.026	0.004/0.01	0.18/0.03	0.002	2.16	$^{\alpha}1\times10^{-5}$	0.016
Sm	0.26	0.009	1.43	2.09	20	0.24	0.024	0.011/0.01	0.05/0.04	0.002	1.68	0.0018	0.014
Gd	0.42	0.020	4.84	2.53	20	0.19	0.018	0.007/0.01	0.04/0.04	0.011	1.46	0.0007	$^{\alpha}1\times10^{-5}$
Tb	0.50	0.030	7.80	2.60	19	0.17	0.019	0.013^{β}/0.02	0.06/0.05	0.004	1.42	$^{\alpha}1\times10^{-5}$	0.017
Dy	0.57	0.043	11.5	2.55	18	0.15	0.018	0.019/0.02	0.29/0.06	0.007	1.26^{β}	0.010	0.015
Y	0.60	0.054	14.1	2.47	17.5	0.14	0.018	0.021/0.04	1.00/0.07	0.021	1.11^{β}	0.007	$^{\alpha}1\times10^{-5}$
Ho	0.62	0.060	15.3	2.41	16.8	0.13	0.018	0.029^{β}/0.04	0.07/0.08	0.010	0.95	$^{\alpha}1\times10^{-5}$	$^{\alpha}1\times10^{-5}$
Er	0.64	0.079	18.8	2.22	15.5	0.12	0.018	0.036/0.07	0.10/0.09	0.020	0.88^{β}	0.012	$^{\alpha}1\times10^{-5}$
Tm	0.64	0.101	21.5	2.00	14.2	0.10	0.018	0.054^{β}/0.10	0.29/0.10	0.026	0.81^{β}	$^{\alpha}1\times10^{-5}$	$^{\alpha}1\times10^{-5}$
Yb	0.64	0.125	23.2	1.79	13	0.09	0.018	0.071/0.13	0.30/0.11	0.026	0.74	0.016	0.017
Lu	0.62	0.149	24.1	1.59	10	0.09	0.018	0.112/0.19	0.33/0.12	0.041	0.54	0.018	0.012
Sr	0.03	0.047	0.02	0.39	1.4	6.65	0.022	0.004/0.002	0.60/0.10	0.0006	0.24	0.003	$^{\alpha}1\times10^{-5}$

Table S4: Sets of static $D^{\mathrm{mineral/melt}}_{\mathrm{REE,Y,Sr}}$ used in this study. Those indicated with * are used in the main modelling, with sources as outlined in Table S3. Those marked with $^+$ are the set of values used in Fig. S10 instead of the dynamic models from Table S3, and shown as red crosses on Fig. 6, and are a set of values from Bédard (2006) for all minerals except olivine, which are from Molendijk et al. (2023) for evolved systems (<10 wt% MgO). If no values labelled $^+$ are given for a certain phase, the values labelled * are used for the results in Fig. S10. $^{\alpha}$no value given, so this number is used instead of zero. $^{\beta}$no value given, so this value is calculated as the average of the two adjacent reported REEs, instead of using zero.

Details of trace element partitioning models used

The trace element partitioning models used for the results presented in the main text are summarised in Table S3. The following discussion outlines the models and also discusses results of comparisons using alternative models for the three key phases: clinopyroxene, amphibole and garnet. For any trace element partitioning parameterisations that use a lattice strain model, ionic radii of each REE^{3+} cation are taken from Shannon (1976) for the relevant coordination number as detailed on a mineral by mineral basis below.

Amphibole

For amphibole, REE and Y partitioning is modelled using the lattice strain model of Shimizu et al. (2017), which was calibrated for basaltic to andesitic systems over 780–1100 °C and 2–25 kbar. Their model is explicitly designed for fractional crystallisation of arc magmas. The lattice strain parameters depend on SiO_2, CaO and TiO_2 in the melt, and the ferromagnesian content of amphibole, with the full lattice strain calculation then introducing temperature as an additional variable (Blundy and Wood, 1994). REEs are taken to be 8-fold coordinated. $D^{\mathrm{amphibole/melt}}_{\mathrm{Sr}}$ is a static value (consistent with observations that it is invariant with melt composition and temperature; Nandedkar et al., 2016), taken as the average from the values reported in the compilation of Bonechi et al. (2023).

As an alternative, Bonechi et al. (2023) presented a multiple linear regression approach, in which $D^{\mathrm{amphibole/melt}}_{\mathrm{REE,Y}}$ is predicted from a combination of pressure, temperature, amphibole Mg# and network-forming cations in the melt. Although this model includes different variables, it is calibrated over a similar pressure, temperature and compositional range as Shimizu et al. (2017), and the two models generally perform comparably (Bonechi et al., 2023). In our calculations, the regression-based approach yields slightly higher $D^{\mathrm{amphibole/melt}}_{\mathrm{La}}$ than the lattice strain model, while predicted $D^{\mathrm{amphibole/melt}}_{\mathrm{Dy}}$ and $D^{\mathrm{amphibole/melt}}_{\mathrm{Yb}}$ are broadly similar between the two (consistent with comparisons by Bonechi et al., 2023; Fig. S5). One notable difference is that low-Ca amphiboles (predicted to form in late-stage, hydrous crystallisation in our crystallisation models) do not exhibit a decrease in $D^{\mathrm{amphibole/melt}}_{\mathrm{REE}}$ using the Bonechi et al. (2023) model (Fig. S5), in contrast to the drop seen (e.g. Fig. S2) when using the Shimizu et al. (2017) lattice strain formulation. Additionally, because the regression model applies different predictor variables to each REE, it tends to produce less smooth REE patterns, with

discontinuities between adjacent elements in REE patterns. Therefore, we use the Shimizu et al. (2017) model for the main modelling here.

Apatite

$D_{\mathrm{REE,Y}}^{\mathrm{apatite/melt}}$ is calculated using the lattice strain model of Jirku et al. (2025). The model depends on temperature, and melt CaO and P_2O_5. REEs are taken to be 9-fold coordinated. Sr partitioning is tied to the value of $D_{\mathrm{Y}}^{\mathrm{apatite/melt}}$, following the observation that the ratio of $D_{\mathrm{Sr}}^{\mathrm{apatite/melt}}$ to $D_{\mathrm{Y}}^{\mathrm{apatite/melt}}$ is not systematically related to melt composition (Nathwani et al., 2020). We use a ratio of 4.3/7.1, taking the values from Prowatke and Klemme (2006) from experiment 61B, an andesitic composition.

Clinopyroxene

Clinopyroxene/melt REE and Y partitioning is calculated using the lattice strain model of Bédard (2014), which is calibrated across a wide melt compositional range from basalts to rhyolites. The model is temperature-dependent, and is calibrated down to 850 °C; below this temperature, we fix $D_{\mathrm{REE,Y}}^{\mathrm{clinopyroxene/melt}}$ to be the value of that at 850 °C to avoid any unexpected behaviours. In the calculation of the ideal site size (r_0) of the M2 site, we identified an error in the cubic coefficient given in Table 2 of Bédard (2014) (given as 2.0599×10^{-3} instead of 2.0599×10^{-4}; following pers. comms. with J. Bedard), and so we use the corrected coefficient here. REEs are taken to be 8-fold coordinated on the M2 site, and 6-fold coordinated on the M1 site. $D_{\mathrm{Sr}}^{\mathrm{clinopyroxene/melt}}$ is calculated using the average of the empirical regressions (eqs. 18–21) presented in Bédard (2014), which depend on MgO, Mg# and total alkalis in the melt, and Mg# of the clinopyroxene. $D_{\mathrm{P}}^{\mathrm{clinopyroxene/melt}}$ is calculated using an empirical regression (eq. 261) presented in Bédard (2014). We use this regression equation, which considers Al_T in clinopyroxene as the variable, because it has the best R^2 of the presented regressions.

As an alternative for $D_{\mathrm{REE,Y}}^{\mathrm{clinopyroxene/melt}}$, we also consider the lattice strain model of Beard et al. (2019), which uses additional experimental data and is designed for tholeiitic basalts, although it extends up to peralkaline phonolites (the latter not being directly relevant here). This model has a complex dependence on clinopyroxene composition (considering an extensive range of cations including Ti, Ca, Mg, Fe^{2+}, Fe^{3+} and Al), as well as depending on temperature and pressure.

At high temperatures, both parameterisations yield similar D_{REE} (Fig. S5). However, the Beard et al. (2019) model predicts a more gradual increase in D with decreasing temperature compared to Bédard (2014). The models diverge considerably at lower temperatures: the Beard et al. (2019) parameterisation predicts that La remains incompatible in late-stage clinopyroxene, and produces $D_{\mathrm{Yb}} > D_{\mathrm{Dy}}$, whereas the Bédard (2014) model indicates that all REEs become compatible and predicts $D_{\mathrm{Dy}} > D_{\mathrm{Yb}}$. The former behaviour occurs because of the strong predicted compatibility of HREE on the small M1 site in Fe-rich clinopyroxene (Beard et al., 2019), with the Fe content of the mineral being an input in the REE partitioning model. The application of each model to the arc systems here carries specific caveats. The Bédard (2014) approach relies on an empirical link between D_{Sm} and temperature, calibrated only down to 850 °C. Although, by contrast, the temperature range modelled here is consistent with the calibration range of Beard et al. (2019), the evolved clinopyroxenes in their dataset are largely from phonolitic systems rather than andesitic to rhyolitic melts. Therefore, it is not clear which predicted partitioning behaviour in evolved arc magmas may be most realistic.

Fluid

We use $D_{\mathrm{REE,Y}}^{\mathrm{fluid/melt}}$ from Yang (2019). We take their 'maximum' values as these relate to a Cl-rich system, given that evolved arc magmas are often chlorine-rich (e.g. Borchardt and Lee, 2024). The study by Yang (2019) includes parameterisations of $D_{\mathrm{REE,Y}}^{\mathrm{fluid/melt}}$ that depend on chlorine molality in the fluid, but since Cl is not part of our model system, we cannot use these parameterisations. Many other studies also present

$D^{fluid/melt}_{REE,Y}$ in variously Cl-bearing systems (e.g. Zajacz et al., 2008; Borchert et al., 2010), which would provide valuable sets of partition coefficients if chlorine is added into this modelling approach in the future. However, for most petrogenetic conditions considered here, a free fluid phase does not exsolve before modelled fractional crystallisation is stopped at 25 % of the system remaining, and so the choice of partition coefficients for fluid does not impact our results.

Garnet

In the results presented in the main text, garnet/melt REE and Y partitioning is modelled using the lattice strain model of Meltzer and Kessel (2020), which is calibrated for hydrous systems between 700–1230 °C, closely matching the temperature range of our modelled fractionation. The lattice strain parameters D_0 and E depend on the ratio of Fe in the garnet relative to the melt, and the melt FeO, SiO_2, Mg# and H_2O, respectively. We use a fixed r_0 value of 0.953, the approximately constant value given in Meltzer and Kessel (2020) for suprasolidus garnets in equilibrium with hydrous melts. Temperature is then introduced as a variable through the lattice strain approach (Blundy and Wood, 1994). REEs are taken to be 8-fold coordinated. However, the Meltzer and Kessel (2020) model was developed for pressures > 15 kbar, higher than those considered in the arc magma modelling here. As an alternative, we also evaluate the lattice strain model of Sun and Liang (2013) because it was calibrated for garnet in equilibrium with basaltic melt. In addition to temperature, this model includes pressure and the grossular component of garnet as inputs. However, the calibration conditions were also designed to be representative of the upper mantle (1325–2300 °C and 24–250 kbar). Although the Sun and Liang (2013) model lies outside the pressure-temperature conditions of our study, it provides a comparison that is designed for basalts, and no alternative parameterisation designed for lower pressures could be found, so we use this as a comparison.

Both models predict similarly low $D^{garnet/melt}_{La}$ (typically < 0.1). However, $D^{garnet/melt}_{Dy}$ and $D^{garnet/melt}_{Yb}$ are extremely large when calculated with the Sun and Liang (2013) model, up to 340 and 1880, respectively, at 10 kbar and 3 wt% H_2O (Fig. S5). These high values result from large strain-free partition coefficients (D_0) which increase with decreasing temperature in the Sun and Liang (2013) parameterisation. Since we are substantially outside the calibrated temperature range, it is not clear if these values are realistic. In cases where the grossular component of garnet decreases substantially and almandine increases during crystallisation (such as at 10 kbar with 4 wt% H_2O), $D^{garnet/melt}_{REE}$ increases accordingly using the Sun and Liang (2013) parameterisation (Fig. S5), in agreement with the result using Meltzer and Kessel (2020), but in this case because grossular component is the compositional variable in the model.

Static values of $D^{garnet/melt}_{Sr}$ are used since no parameterisations could be found. For the modelling in the main text, we use a value of 0.031, which comes from a tonalitic bulk composition (Jenner et al., 1993). As a comparison, when we consider the alternative Sun and Liang (2013) model for REE and Y partitioning, we also vary $D^{garnet/melt}_{Sr}$, using a value of 0.014, the lowest literature value as given in Jenner et al. (1993), and consistent with the value also proposed by Bédard (2006).

Magnetite and spinel

We consider magnetite and spinel with the same $D^{mineral/melt}$. REE and Y partitioning is calculated following the lattice strain model of Sievwright et al. (2020) designed for magnetite, which is temperature-dependent. We use the values for the lattice strain parameters (E, r_0, D_0) given for ΔFMQ = 0. REEs are taken to be 6-fold coordinated.

Olivine

Olivine/melt REE, Y, Sr and P partitioning is calculated using a regression approach from Bédard (2005), with melt MgO as the variable. $D_{\mathrm{Sr}}^{\mathrm{olivine/melt}}$ is fixed below 2.7 wt% MgO (Bédard, 2005). Equations for $D_{\mathrm{Pr,Tm}}^{\mathrm{olivine/melt}}$ are not provided, and so to avoid unexpected behaviour in REE results, we calculate each as the average of the two adjacent REEs (Ce and Nd for Pr, Er and Yb for Tm). The coefficients in the regression for $D_{\mathrm{P}}^{\mathrm{olivine/melt}}$ vary depending on melt MgO content, and $D_{\mathrm{P}}^{\mathrm{olivine/melt}}$ is fixed at < 2 wt% MgO.

Orthopyroxene

The lattice strain model of Bédard (2025) is used for $D_{\mathrm{REE,Y}}^{\mathrm{orthopyroxene/melt}}$. This model considers melt MgO content as the variable for the lattice strain parameters (E, D_0, r_0), in addition to the temperature dependence that is then inherent to a lattice strain calculation (Blundy and Wood, 1994). REEs are taken to be 6-fold coordinated. Sr partitioning is calculated using an empirical regression (eq. 29) with melt MgO/(MgO + FeO_T) (wt%) as the variable (Bédard, 2025). $D_{\mathrm{P}}^{\mathrm{orthopyroxene/melt}}$ is also calculated using an empirical regression (eq. 133; Bédard, 2025) that is dependent on melt SiO_2. This regression is used because it has the best R^2 of the reported regressions.

Plagioclase feldspar

$D_{\mathrm{REE,Y}}^{\mathrm{plagioclase/melt}}$ is calculated using the lattice strain model of Bédard (2023). The lattice strain parameters are dependent on anorthite content (calculated using the hydrous set of parameters from Bédard, 2023), and temperature-dependence is introduced through the lattice strain approach (Blundy and Wood, 1994). REEs are taken to be 8-fold coordinated. $D_{\mathrm{Sr}}^{\mathrm{plagioclase/melt}}$ is calculated from the average of the regression equations (eqs. 84–95) which consider anorthite content, orthoclase content, melt SiO_2, MgO, Mg# and total alkalies. $D_{\mathrm{P}}^{\mathrm{plagioclase/melt}}$ is calculated from eq. 121 in Bédard (2023), which depends on melt SiO_2. This equation is used because it has the best R^2 of the provided regressions.

Supplementary figures

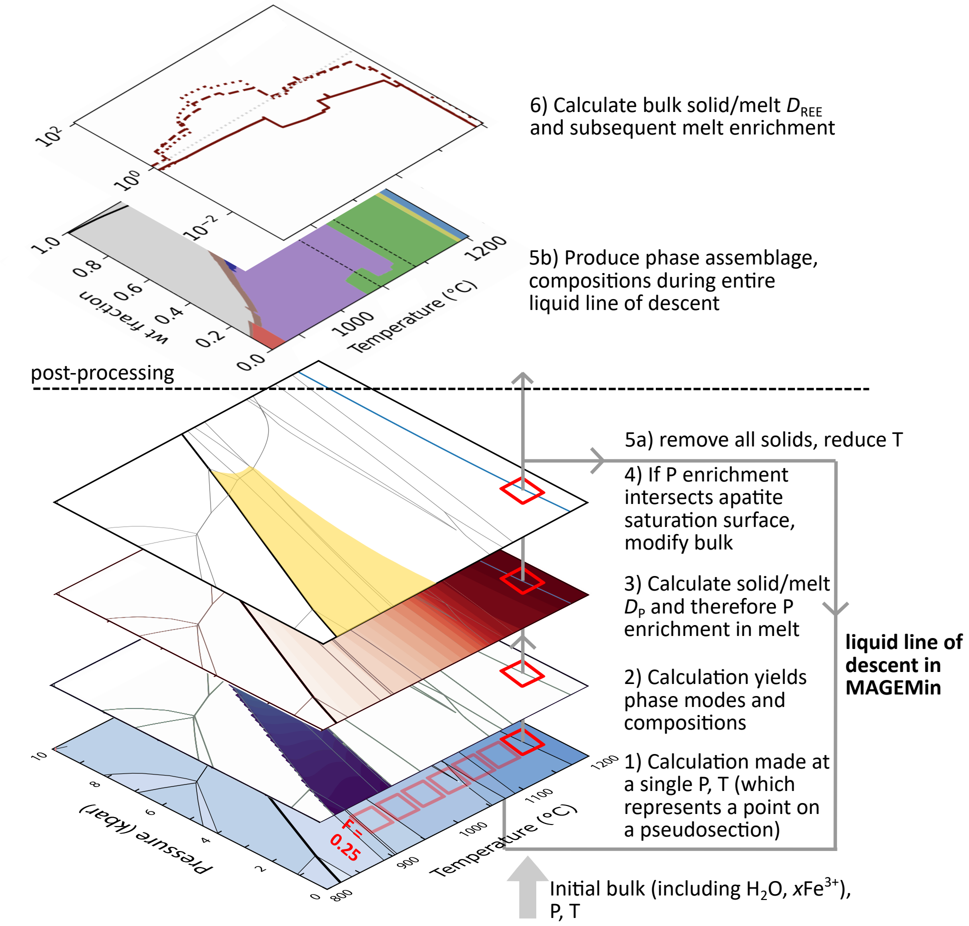

Figure S1: Schematic of the modelling approach, highlighting stages of the calculation that are performed in MAGEMin to calculate the liquid line of descent versus those that are performed subsequently. The liquid line of descent is calculated until F (weight fraction of system remaining) is 0.25. $D_{\mathrm{P,REE}}$ refer to bulk $D^{\mathrm{solid/melt}}_{\mathrm{P,REE}}$, calculated using each $D^{\mathrm{mineral/melt}}_{\mathrm{P,REE}}$ and the abundance of each mineral at a given point.

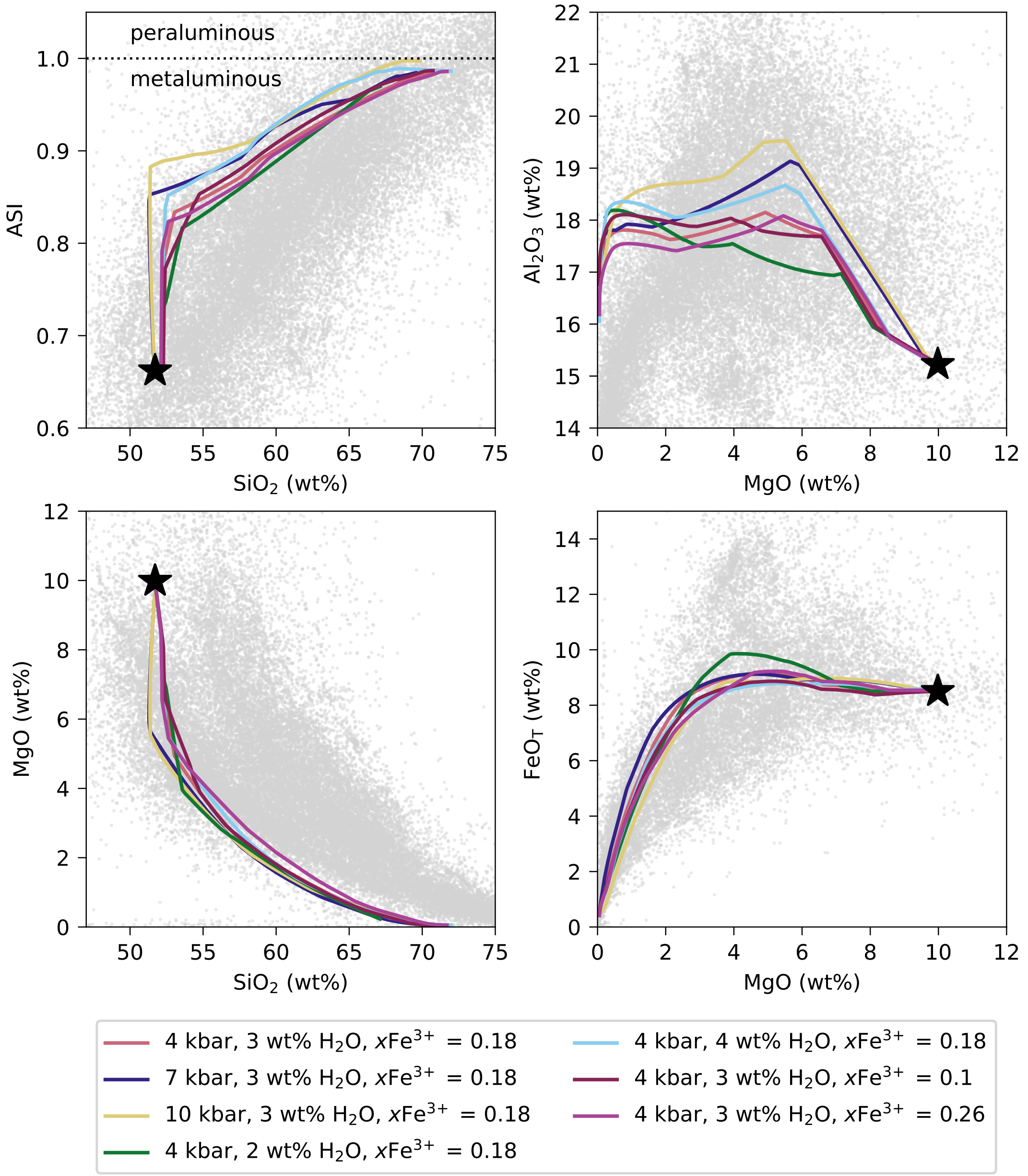

Figure S2: Modelled melt compositions during fractional crystallisation for a variety of petrogenetic conditions. ASI = molar $Al_2O_3/(CaO + Na_2O + K_2O)$. The star shows the average primitive arc composition used (Tables S1, S2). Background dataset is the ArcMetals database (Barber et al., 2021).

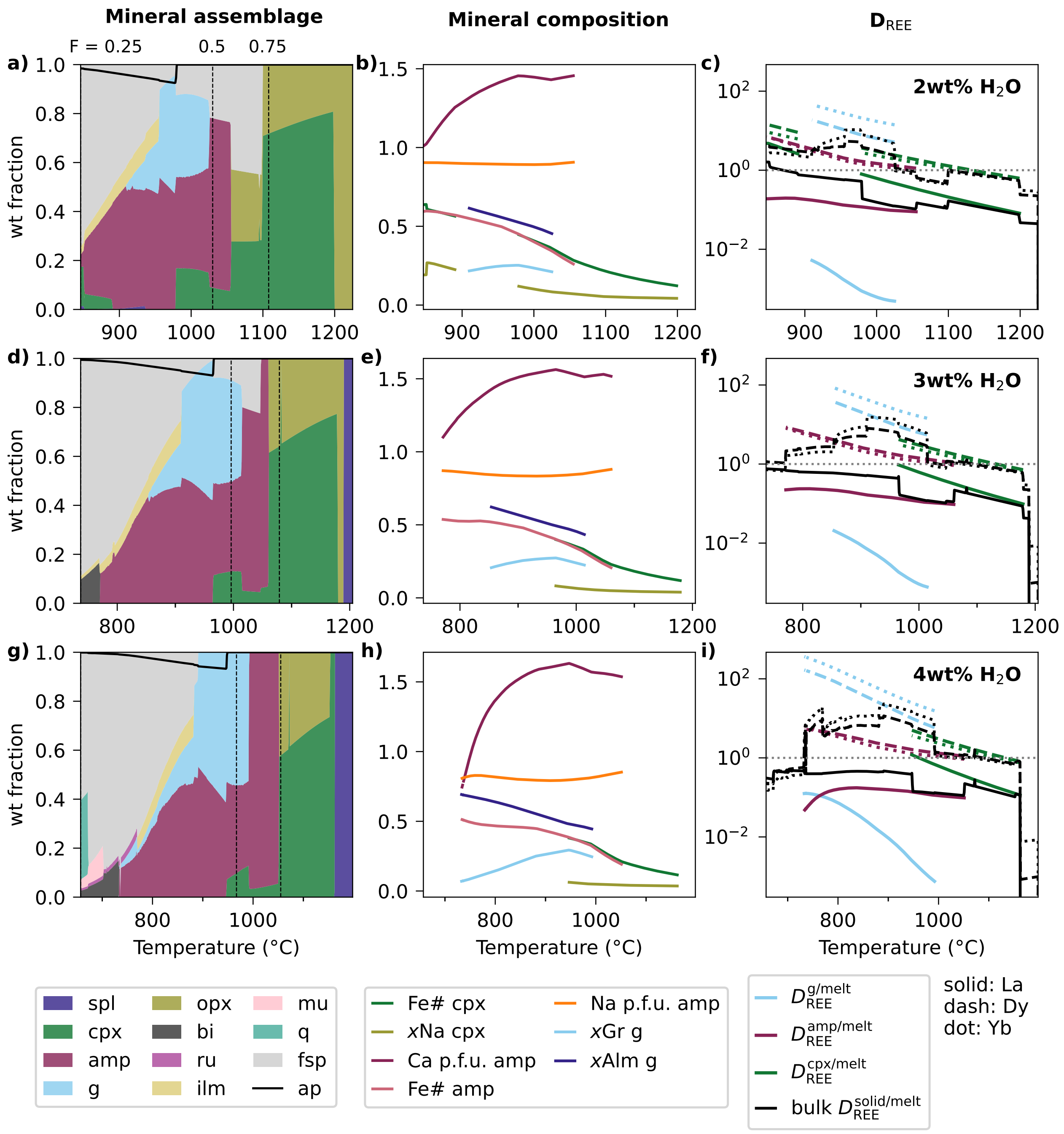

Figure S3: Instantaneous mineral assemblages (panels a, d, g), mineral compositions (panels b, e, h) and REE partitioning results (panels c, f, i) during fractional crystallisation of an average primitive arc magma at 10 kbar and xFe^{3+} = 0.18 with 2 (a–c), 3 (d–f) and 4 (g–i) wt % initial H_2O. Panels d–f are identical to panels g–i in Fig. 5 of the main text, but are included again for easy comparison. Compositions and $D^{\mathrm{mineral/melt}}$ are only shown for the minerals with key controls on trace element behaviour (amphibole, garnet, clinopyroxene). In panels a, d and g, vertical dashed lines show 75 and 50 wt% of the system remaining, and the x-axis is cut off at 25 % remaining. Abbreviations as in Figs. 1, 2 & 5 of the main text, and additionally mu = muscovite.

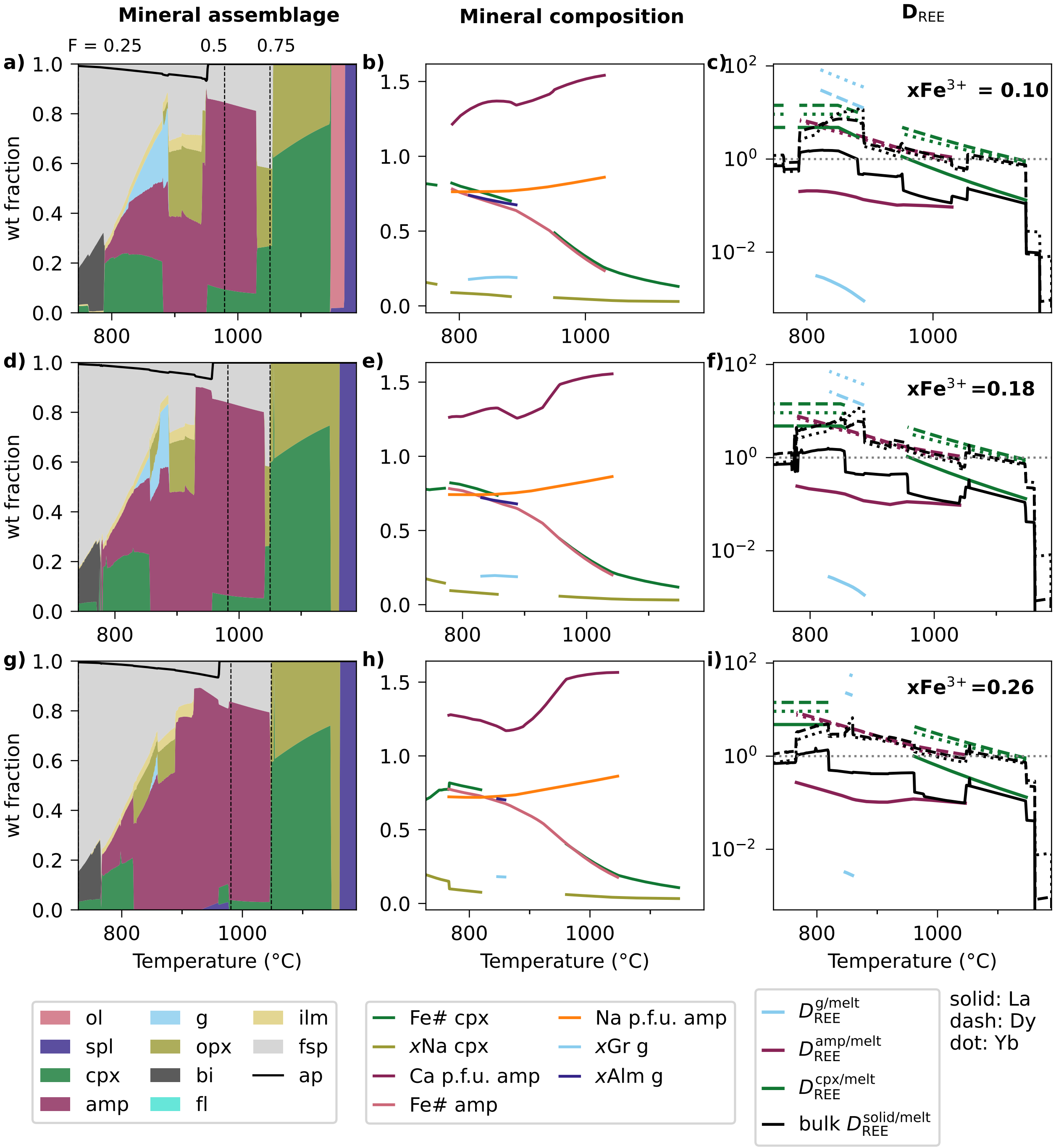

Figure S4: Instantaneous mineral assemblages (panels a, d, g), mineral compositions (panels b, e, h) and REE partitioning results (panels c, f, i) during fractional crystallisation of an average primitive arc magma at 7 kbar and 3 wt% initial H_2O with $xFe^{3+} = 0.10$ (a–c), 0.18 (d–f) and 0.26 (g–i). Panels d–f are identical to panels g–i in Fig. 5 of the main text, but are included again for easy comparison. Compositions and $D^{\mathrm{mineral/melt}}$ only shown for the minerals with key controls on trace element behaviour (amphibole, garnet, clinopyroxene). In panels a, d and g, vertical dashed lines show 75 and 50 wt% of the system remaining, and the x-axis is cut off at 25 % remaining. Abbreviations as in Figs. 1, 2 & 5 of the main text.

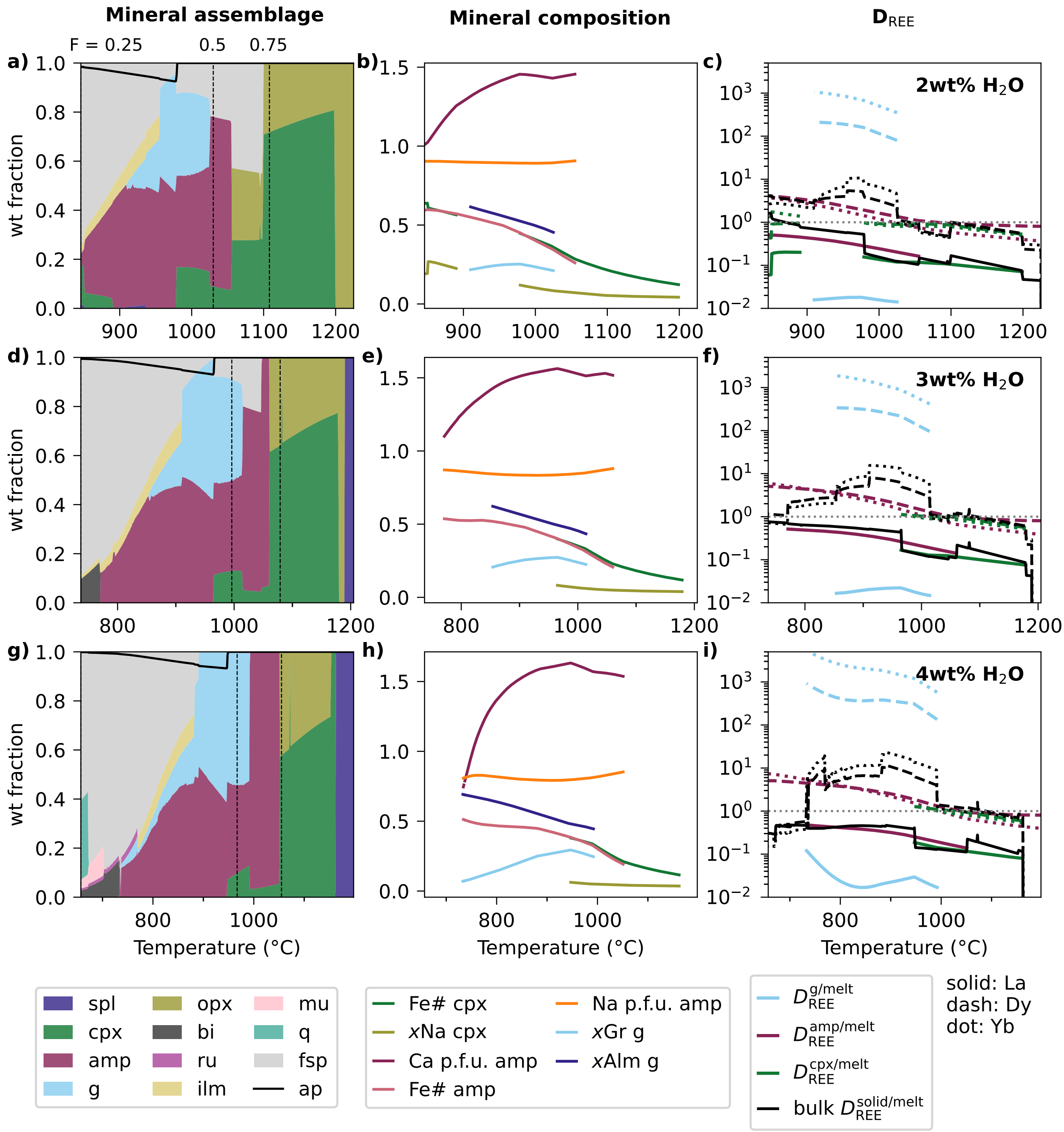

Figure S5: Comparison to Fig. S3 (10 kbar, xFe^{3+}, variable initial H_2O) using alternative $D_{\text{REE}}^{\text{mineral/melt}}$ models for clinopyroxene, amphibole and garnet (Beard et al., 2019; Bonechi et al., 2023; Sun and Liang, 2013). All other $D^{\text{mineral/melt}}$ are as per the modelling presented in the main text. Details are given in the supplementary text. Instantaneous mineral assemblages and mineral compositions as per Fig. S3.

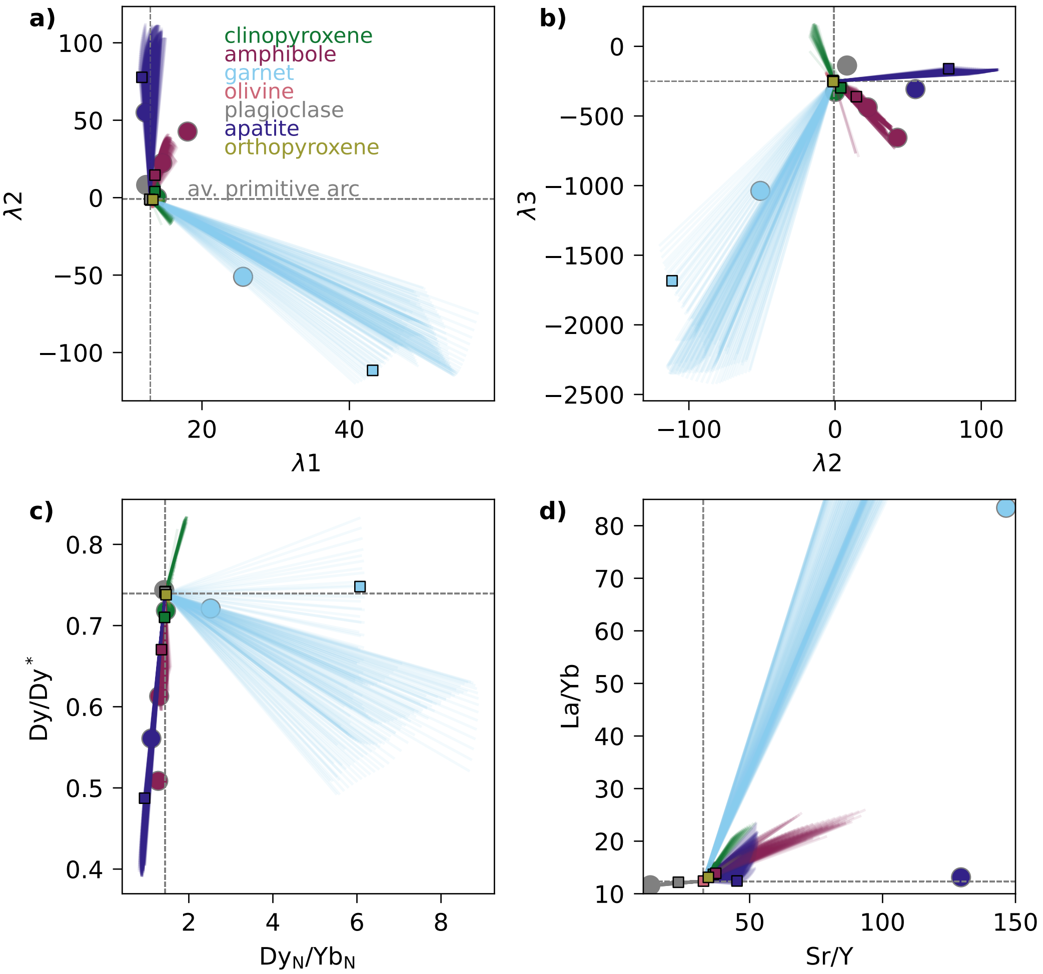

Figure S6: Version of Fig. 8 of main text with alternative D parameterisations for amphibole (Bonechi et al., 2023), clinopyroxene (Beard et al., 2019) and garnet (Sun and Liang, 2013). See Fig. S5 caption and supplementary text for further details on the D models, and Fig. 8 caption of the main text for the figure details. The vectors show the effect of crystallising 20 % of each mineral (which each have varying $D_{\mathrm{REE}}^{\mathrm{mineral/melt}}$ during fractionation depending on temperature and composition) for every 5 °C of cooling in the modelled arc magma evolution. For reference, small squares show the highest temperature (i.e. initial) vector for the 10 kbar, 3 wt% initial H_2O, $x\mathrm{Fe}^{3+} = 0.18$ case for all phases, except olivine which is shown for the 4 kbar case. Circles show the result of using static D values from Bédard (2006) for all phases except amphibole (as used for modelling trace element evolution by Tatnell et al., 2023).

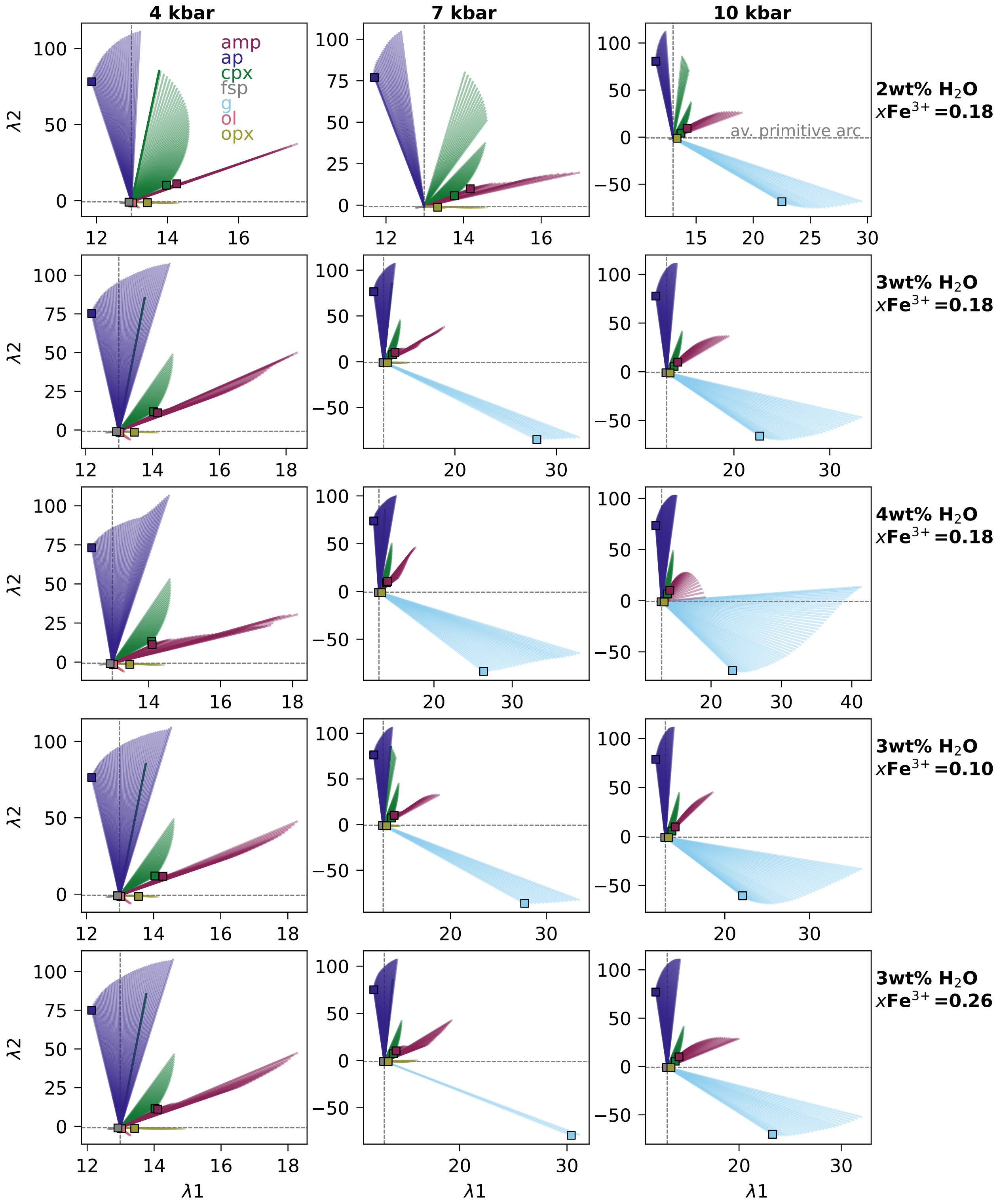

Figure S7: Version of panel a of Fig. 8 in the main text showing each set of vectors separated by petrogenetic scenario. The given water content refers to the initial conditions of the fractional crystallisation model. The combination of ten of these fifteen panels (4 and 10 kbar results only) is shown in Fig. 8a of the main text, and an equivalent methodology was applied for all the other trace element ratios considered.

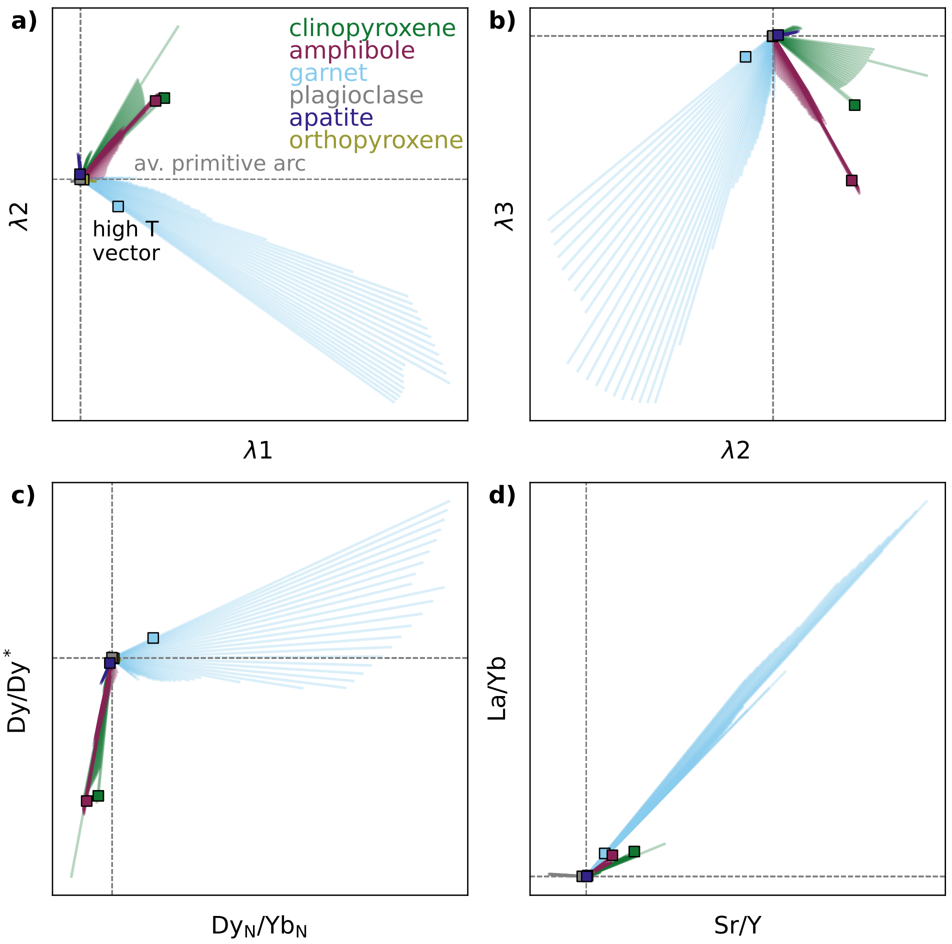

Figure S8: Equivalent of Fig. 9 of the main text but at 10 kbar with $xFe^{3+} = 0.18$ and initial 4 wt% H_2O, showing mineral vectors scaled by the contribution of the mineral to the cumulative mineral assemblage.

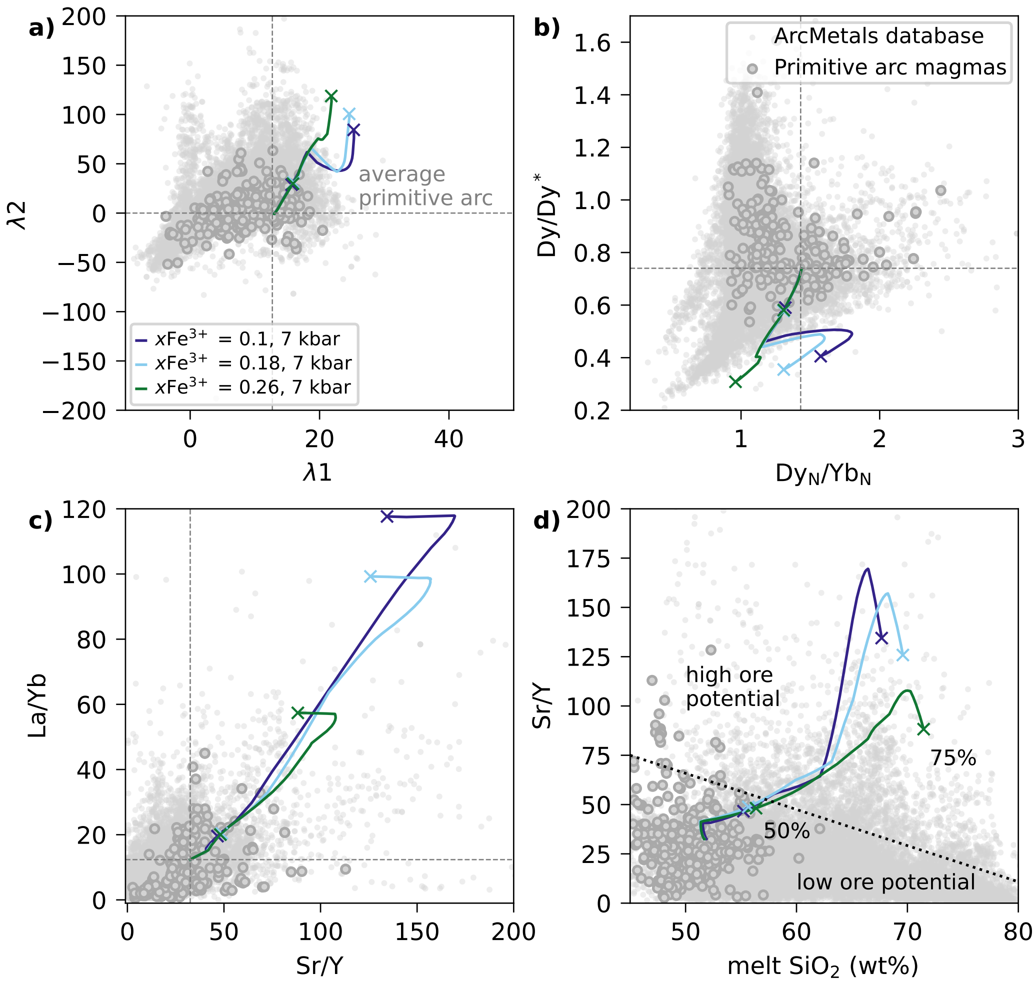

Figure S9: Modelled evolution of the trace element composition of a fractionating melt with varying $x\mathrm{Fe}^{3+}$. All calculations at 7 kbar with 3 wt% initial H_2O. Background dataset is the ArcMetals database (Barber et al., 2021), with outlined samples showing the primitive arc magmas identified by Tatnell et al. (2023). In d), the dividing line between 'high ore potential' and 'low ore potential' magmas is from Loucks (2014). Crosses mark 50 and 75 wt% fractional crystallisation.

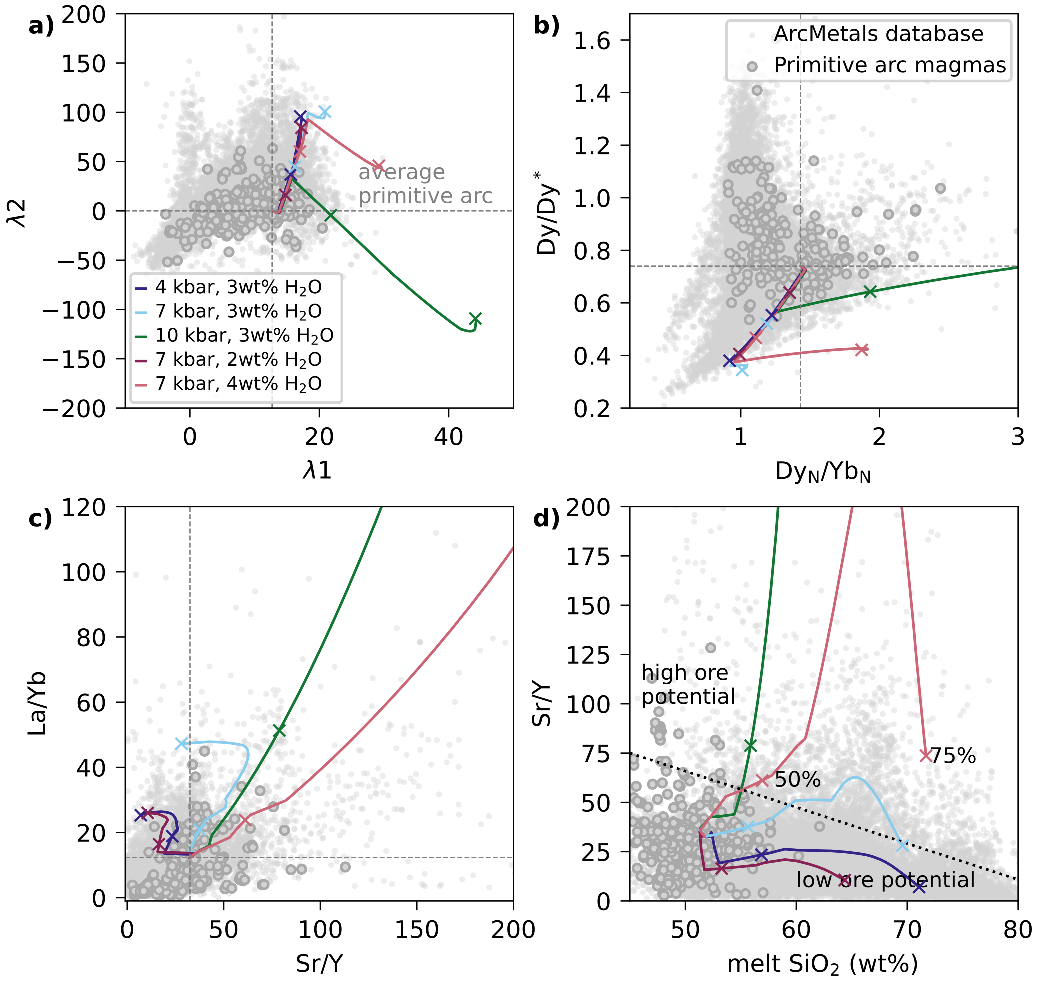

Figure S10: Version of Fig. 10 from main text but using a static $D^{\text{mineral/melt}}$ suite, mostly from Bédard (2006), following Tatnell et al. (2023). See Table S4 for details. Background dataset is the ArcMetals database (Barber et al., 2021), with outlined samples showing the primitive arc magmas identified by Tatnell et al. (2023). In d), the dividing line between 'high ore potential' and 'low ore potential' magmas is from Loucks (2014). Crosses mark 50 and 75 wt% fractional crystallisation. The dark and light blue and dark pink lines in panel d are shown as dashed lines of the same colour in Fig. 10 of the main text.

References

N. D. Barber, M. Edmonds, F. Jenner, A. Audétat, and H. Williams. Amphibole control on copper systematics in arcs: Insights from the analysis of global datasets. *Geochimica et Cosmochimica Acta*, 307:192–211, 2021.

C. D. Beard, V. J. van Hinsberg, J. Stix, and M. Wilke. Clinopyroxene/melt trace element partitioning in sodic alkaline magmas. *Journal of Petrology*, 60(9):1797–1823, 2019.

J. H. Bédard. Partitioning coefficients between olivine and silicate melts. *Lithos*, 83(3-4):394–419, 2005.

J. H. Bédard. A catalytic delamination-driven model for coupled genesis of Archaean crust and sub-continental lithospheric mantle. *Geochimica et Cosmochimica Acta*, 70(5):1188–1214, 2006.

J. H. Bédard. Parameterizations of calcic clinopyroxene—melt trace element partition coefficients. *Geochemistry, Geophysics, Geosystems*, 15(2):303–336, 2014.

J. H. Bédard. Trace element partitioning coefficients between terrestrial silicate melts and plagioclase feldspar: Improved and simplified parameters. *Geochimica et Cosmochimica Acta*, 350:69–86, 2023.

J. H. Bédard. Trace element partitioning coefficients between orthopyroxene and melt: Parameterizations of D variations and an improved Lattice Strain Model for rare earth elements. *Chemical Geology*, 681:122710, 2025.

J. Blundy and B. Wood. Prediction of crystal–melt partition coefficients from elastic moduli. *Nature*, 372(6505): 452–454, 1994.

B. Bonechi, A. Fabbrizio, C. Perinelli, M. Gaeta, and M. Petrelli. Experimental investigation of trace element partitioning between amphibole and alkali basaltic melt: Toward a more general partitioning model with implications for amphibole fractionation at deep crustal levels. *American Mineralogist*, 108(9):1678–1691, 2023.

J. S. Borchardt and C.-T. A. Lee. The chlorine evolution of arc magmas and the crustal water filter. *Earth and Planetary Science Letters*, 648:119048, 2024.

M. Borchert. *Interactions between aqueous fluids and silicate melts*. PhD thesis, Universität Potsdam, 2009.

M. Borchert, M. Wilke, C. Schmidt, J. Cauzid, and R. Tucoulou. Partitioning of Ba, La, Yb and Y between haplogranitic melts and aqueous solutions: An experimental study. *Chemical Geology*, 276(3-4):225–240, 2010.

S. F. Foley, M. G. Barth, and G. A. Jenner. Rutile/melt partition coefficients for trace elements and an assessment of the influence of rutile on the trace element characteristics of subduction zone magmas. *Geochimica et Cosmochimica Acta*, 64(5):933–938, 2000.

G. A. Jenner, S. F. Foley, S. E. Jackson, T. H. Green, B. J. Fryer, and H. P. Longerich. Determination of partition coefficients for trace elements in high pressure-temperature experimental run products by laser ablation microprobe-inductively coupled plasma-mass spectrometry (LAM-ICP-MS). *Geochimica et Cosmochimica Acta*, 57(23-24):5099–5103, 1993.

M. Jirku, V. Špillar, and A. Fabbrizio. Lattice strain model for rare earth element partitioning between apatite and silicate melt: effect of apatite/melt composition and temperature with implications for lunar basalts. *Mineralogy and Petrology*, 119(1):1–19, 2025.

S. Klemme, S. Prowatke, K. Hametner, and D. Günther. Partitioning of trace elements between rutile and silicate melts: implications for subduction zones. *Geochimica et Cosmochimica Acta*, 69(9):2361–2371, 2005.

S. Klemme, D. Günther, K. Hametner, S. Prowatke, and T. Zack. The partitioning of trace elements between ilmenite, ulvospinel, armalcolite and silicate melts with implications for the early differentiation of the moon. *Chemical Geology*, 234(3-4):251–263, 2006.

R. R. Loucks. Distinctive composition of copper-ore-forming arc magmas. *Australian Journal of Earth Sciences*, 61(1):5–16, 2014.

A. Meltzer and R. Kessel. Modelling garnet-fluid partitioning in H_2O-bearing systems: a preliminary statistical attempt to extend the crystal lattice-strain theory to hydrous systems. *Contributions to Mineralogy and Petrology*, 175(8):80, 2020.

S. M. Molendijk, O. Namur, P. R. Mason, B. Dubacq, B. Smets, D. A. Neave, and B. Charlier. Trace element partitioning in silica-undersaturated alkaline magmatic systems. *Geochimica et Cosmochimica Acta*, 346: 29–53, 2023.

R. H. Nandedkar, P. Ulmer, and O. Müntener. Fractional crystallization of primitive, hydrous arc magmas: an experimental study at 0.7 GPa. *Contributions to Mineralogy and Petrology*, 167(6):1015, 2014.

R. H. Nandedkar, N. Hürlimann, P. Ulmer, and O. Müntener. Amphibole–melt trace element partitioning of fractionating calc-alkaline magmas in the lower crust: an experimental study. *Contributions to Mineralogy and Petrology*, 171(8):71, 2016.

W. P. Nash and H. R. Crecraft. Partition coefficients for trace elements in silicic magmas. *Geochimica et Cosmochimica Acta*, 49(11):2309–2322, 1985.

C. L. Nathwani, M. A. Loader, J. J. Wilkinson, Y. Buret, R. H. Sievwright, and P. Hollings. Multi-stage arc magma evolution recorded by apatite in volcanic rocks. *Geology*, 48(4):323–327, 2020.

S. Prowatke and S. Klemme. Trace element partitioning between apatite and silicate melts. *Geochimica et Cosmochimica Acta*, 70(17):4513–4527, 2006.

R. D. Shannon. Revised effective ionic radii and systematic studies of interatomic distances in halides and chalcogenides. *Foundations of Crystallography*, 32(5):751–767, 1976.

K. Shepherd, O. Namur, M. J. Toplis, J.-L. Devidal, and B. Charlier. Trace element partitioning between clinopyroxene, magnetite, ilmenite and ferrobasaltic to dacitic magmas: an experimental study on the role of oxygen fugacity and melt composition. *Contributions to Mineralogy and Petrology*, 177(9):90, 2022.

K. Shimizu, Y. Liang, C. Sun, C. R. Jackson, and A. E. Saal. Parameterized lattice strain models for REE partitioning between amphibole and silicate melt. *American Mineralogist*, 102(11):2254–2267, 2017.

R. H. Sievwright, H. S. C. O'Neill, J. Tolley, J. J. Wilkinson, and A. J. Berry. Diffusion and partition coefficients of minor and trace elements in magnetite as a function of oxygen fugacity at 1150°C. *Contributions to Mineralogy and Petrology*, 175:1–21, 2020.

C. Sun and Y. Liang. The importance of crystal chemistry on REE partitioning between mantle minerals (garnet, clinopyroxene, orthopyroxene, and olivine) and basaltic melts. *Chemical Geology*, 358:23–36, 2013.

L. Tatnell, M. Anenburg, and R. Loucks. Porphyry copper deposit formation: identifying garnet and amphibole fractionation with REE pattern curvature modeling. *Geophysical Research Letters*, 50(14):e2023GL103525, 2023.

P. Ulmer, R. Kaegi, and O. Müntener. Experimentally derived intermediate to silica-rich arc magmas by fractional and equilibrium crystallization at 1.0 GPa: an evaluation of phase relationships, compositions, liquid lines of descent and oxygen fugacity. *Journal of Petrology*, 59(1):11–58, 2018.

O. M. Weller, T. J. B. Holland, C. R. Soderman, E. C. R. Green, R. Powell, C. D. Beard, and N. Riel. New thermodynamic models for anhydrous alkaline-silicate magmatic systems. *Journal of Petrology*, 65(10): egae098, 2024.

P. Were and H. Keppler. Trace element fractionation between biotite, allanite, and granitic melt. *Contributions to Mineralogy and Petrology*, 176(9):74, 2021.

X.-M. Yang. Using Rare Earth Elements (REE) to decipher the origin of ore fluids associated with granite intrusions. *Minerals*, 9(7):426, 2019.

Z. Zajacz, W. E. Halter, T. Pettke, and M. Guillong. Determination of fluid/melt partition coefficients by LA-ICPMS analysis of co-existing fluid and silicate melt inclusions: Controls on element partitioning. *Geochimica et Cosmochimica Acta*, 72(8):2169–2197, 2008.
